# Range of Movement in Ray I of Manus and Pes and the Prehensility of the Autopodia in the Early Permian to Late Cretaceous Non-Anomodont Synapsida

**DOI:** 10.1371/journal.pone.0113911

**Published:** 2014-12-17

**Authors:** Susanna B. Kümmell, Eberhard Frey

**Affiliations:** 1 Institute of Evolutionary Biology and Morphology, Center for Biomedical Education and Research, Faculty of Health, School of Medicine, University Witten/Herdecke, Witten, Germany; 2 Staatliches Museum für Naturkunde Karlsruhe, Karlsruhe, Germany; Raymond M. Alf Museum of Paleontology, United States of America

## Abstract

The mobility of ray I was analysed in seventy-eight Early Permian to Late Cretaceous specimens of non-mammalian Synapsida and one extant mammal. In all non-mammaliamorph Synapsida investigated, ray I formed a digital arcade. The first phalanx was maximally extendable to the zero position in the metapodiophalangeal joint I. Metapodiale I was the functional equivalent to a basal phalanx of digits II–V. In contrast, there was no digital arcade in ray I in Mesozoic Mammaliamorpha. Phalanx 1 I was dorsally extendable and metapodiale I was functionally part of the metapodium. During the propulsion phase, autopodial rotation occurred in the majority of Synapsida with abducted limb posture. Regarding ray I, the reduction of autopodial rotation can be estimated, e.g., from the decrease of lateral rotation and medial abduction of the first phalanx in the metapodiophalangeal joint I. Autopodial rotation was high in *Titanophoneus* and reduced in derived Cynodontia. In Mammaliamorpha the mobility of the first ray suggests autopodial rolling in an approximately anterior direction. Most non-mammaliamorph Therapsida and probably some Mesozoic Mammaliamorpha had prehensile autopodia with an opposable ray I. In forms with a pronounced relief of the respective joints, ray I could be opposed to 90° against ray III. A strong transverse arch in the row of distalia supported the opposition movement of ray I and resulted in a convergence of the claws of digits II–V just by flexing those digits. A tight articular coherence in the digital joints of digits II–V during strong flexion supported a firm grip capacity. Usually the grip capacity was more pronounced in the manus than in the pes. Prehensile autopodia of carnivorous Therapsida may have been utilized to hold prey while biting, thus helping to avoid fractures of the laterally compressed fangs.

## Introduction

The mobility of ray I in the manus and pes of the Synapsida that lived from the Early Permian until the Late Cretaceous has not yet been investigated systematically. Many studies have been published on the morphology of autopodial ray I for individual specimens (e.g. [1]–[16]), but a systematic analysis of the movement capacity of autopodial ray I in general has not yet been published. Here, the anatomy of the autopodial ray I of non-anomodont Synapsida is described. Furthermore, the range of movement is reconstructed, and the evolution of this enigmatic structure is discussed for the first time. The standard position of ray I, the movement of ray I during the propulsion phase, as well as the presence and functionality of a prehensile manus and pes are key aspects of this paper. Part of this study was originally completed as a topic of a German-language Ph.D. dissertation [17], but is here expanded by the investigation of additional material with well preserved first rays and by a reinvestigation of specimens in which the first ray has been further prepared since the earlier description.

## Abbreviations

### Museums and Institutions

AMNH, American Museum of Natural History, New York, USA

BP, Bernard Price Institute, ESI Evolutionary Studies Institute, Johannesburg, South Africa

CAGS, Chinese Academy of Geological Science, Beijing, China

CAMZM, University Museum of Zoology, Cambridge, United Kingdom

CGS, Council for Geosciences, Pretoria, South Africa

GMV, National Geological Museum of China, Beijing, China

GPIT, Paleontology Department and Museum, Institute of Geosciences, Eberhard Karls University, Tübingen, Germany

IVPP, Institute for Vertebrate Palaeontology, Beijing, China

MCZ, Museum of Comparative Zoology, Harvard University, Cambridge MA, USA

NHMUK, Natural History Museum, London, United Kingdom

NMQR, National Museum, Bloemfontein, South Africa

PIN, Paleontological Institute, Moscow, Russia

PSS MAE, Paleontological and Stratigraphy Branch (Geological Institute), Mongolian Academy of Sciences, and American Museum of Natural History Expeditions

RC, Rubidge Collection, Wellwood, Graaff-Reinet, South Africa

SAM, South African Museum, Cape Town, South Africa

SGP, Sino-German Project, Tübingen, Germany

TM, Ditsong, National Museum of Natural History, Pretoria, South Africa

TSK, T. S. Kemp Collection Oxford, University Museum, Oxford, United Kingdom

ZFMK, Zoological Research Museum Alexander Koenig, Bonn, Germany

ZPAL, Institute of Paleobiology of the Polish Academy of Sciences, Warsaw, Poland

## Materials and Methods

### 1 Fossil and Extant Material

The biometry of the autopodia of 78 Early Permian to Late Cretaceous specimens from 44 different genera was analysed, including three non-therapsid Synapsida, 27 non-mammaliamorph Therapsida and 14 Mesozoic Mammaliamorpha. Anomodontia were excluded from this study because of the peculiar construction of their autopodia. Our investigations on the anomodont ray I will be published in a separate paper. The autopodia of *Didelphis virginiana* were measured for comparison with the fossil material. In most cases the original fossils were measured. Some measurements were taken from casts or from the literature, and are specified in the following list.

#### List of investigated fossils and one extant mammal

**EUPELYCOSAURIA**: *Edaphosaurus boanerges* NHMUK R 9204 (cast), *Ophiacodon navajovicus* AMNH 4776, 4781, *Dimetrodon* CAMZM T 857 (original and cast), AMNH 24810 (cast), *Dimetrodon teutonis* MNG 10654.

**BIARMOSUCHIA**: *Biarmosuchus tener* PIN 1758/320, *Hipposaurus boonstrai* SAM-PK-8950.

**DINOCEPHALIA**: *Anteosaurus magnificus* SAM-PK-4323, *Titanophoneus* potens PIN 157/1.

**GORGONOPSIA**: *Viatkogorgon ivakhnenkoi* PIN 2212/61, *Gorgonops torvus* BP/1/4089, ?*Gorgonops* SAM-PK-K7580, *Arctognathus curvimola* SAM-PK-3329, *Aelurognathus tigriceps* SAM-PK-2342, *Aelurognathus microdon* SAM-PK-9344, *Rubidgea* BP/1/2167, *cf*. *Rubidgea* BP/1/1210, Gorgonopsia indet. BP/1/4259, BP/1/600, CGS CM 86–471, SAM-PK-10315, SAM-PK-K4441.

**THEROCEPHALIA**: *Glanosuchus macrops* SAM-PK-K7809, *Glanosuchus* SAM-PK-12051, CGS RS424, Therocephalia indet. NMQR 3530, *Theriognathus microps* NHMUK R 5694, BP/1/182, *Theriognathus* NMQR 3375, *Olivierosuchus paringtoni* BP/1/3849, *Ictidosuchoides* BP/1/4092, SAM-PK-K10704, *Ictidosuchoides longiceps* CGS CM86-655, *Ictidosuchops intermedius* BP/1/3155, *Regisaurus* BP/1/3973, Regisauridae indet. CAMZM T 837, *Zorillodontops gracilis* SAM-PK-K1392, *Tetracynodon darti* BP/1/2710, *Ericiolacerta parva* CAMZM T 369, *Bauria cynops* CAMZM T 373 (cast), *Aelurosuchus* SAM-PK-5875, *Microgomphodon oligocynus* SAM-PK-K10160, Therocephalia indet. BP/1/5898, ?Therocephalia TM 4696.

**Non-mammaliamorph CYNODONTIA**: *Procynosuchus delaharpeae* BP/1/591, TSK 34, RC92, *Galesaurus planiceps* BP/1/4506, BP/1/2513a,b, BP/1/4637, SAM-PK-K10468, SAM-PK-K10465 (photos), *Thrinaxodon liorhinus* BP/1/2776, BP/1/1730, BP/1/1737, BP/1/5558 (scan), *Diademodon mastacus* BP/1/3756, *Diademodon* NHMUK R-3581, Cynodontia indet. BP/1/4535, *?Scalenodon* NHMUK R 9391, *Chiniquodon theotonicus* MCZ 3781, *Chinquodon* PVL 3820 (photo).

**MAMMALIAMORPHA**: *Oligokyphus* NHMUK R 7515, 7516, *Eozostrodon parvus* CAMZM Eo PC, *Erythrotherium parringtoni* SAM-PK-K359, *Megazostrodon rudnerae* NHMUK M 26407, *Jeholodens jenkinsi* GMV 2139a (original and cast), *Gobiconodon ostromi* MCZ 19860, *Kryptobaatar dashzevegi* ZPAL MgM-I/41 (data from Kielan-Jaworowska and Gambaryan 1994), *?Eucosmodon* AMNH 16325, *Zhangheotherium quinquecuspidens* IVPP V7466, CAGS-IG-97.07352 (cast), *Henkelotherium guimarotae* Gui Mam 138/76, *Sinodelphys szalayi* CAGS00-IG03 (cast), *Asiatherium reshetovi* PIRAS 3907 (cast), *Eomaia scansoria* CAGS01-IG-1a (cast), *Zalambdalestes lechei* ZPAL MgM-I/43 (data from Kielan-Jaworowska 1978).

**EXTANT MAMMAL**: *Didelphis virginiana* CAMZM A4.5/2

**Figure 1:**
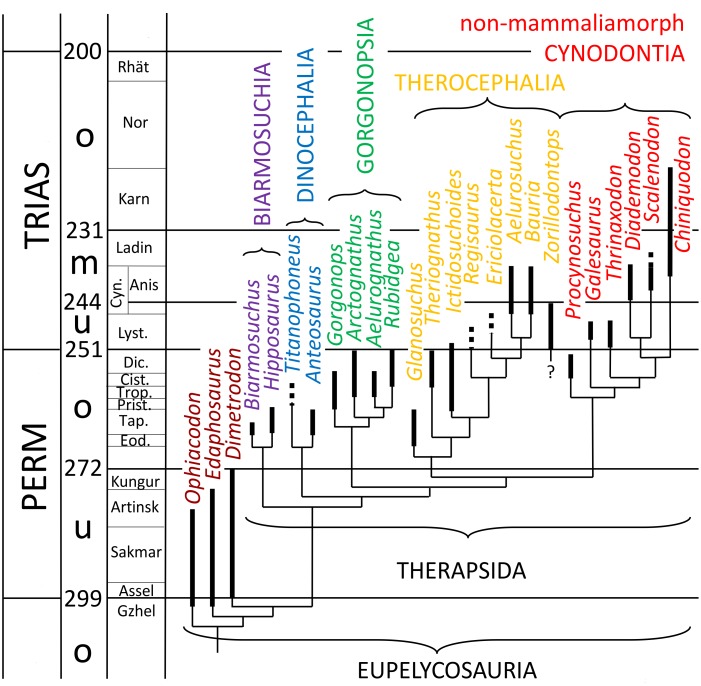
Cladogram of the fossil non-mammaliamorph Synapsida mentioned in the text. The data were taken from [17]–[19] and Fernando Abdala (pers. comm. 2012).

**Figure 2:**
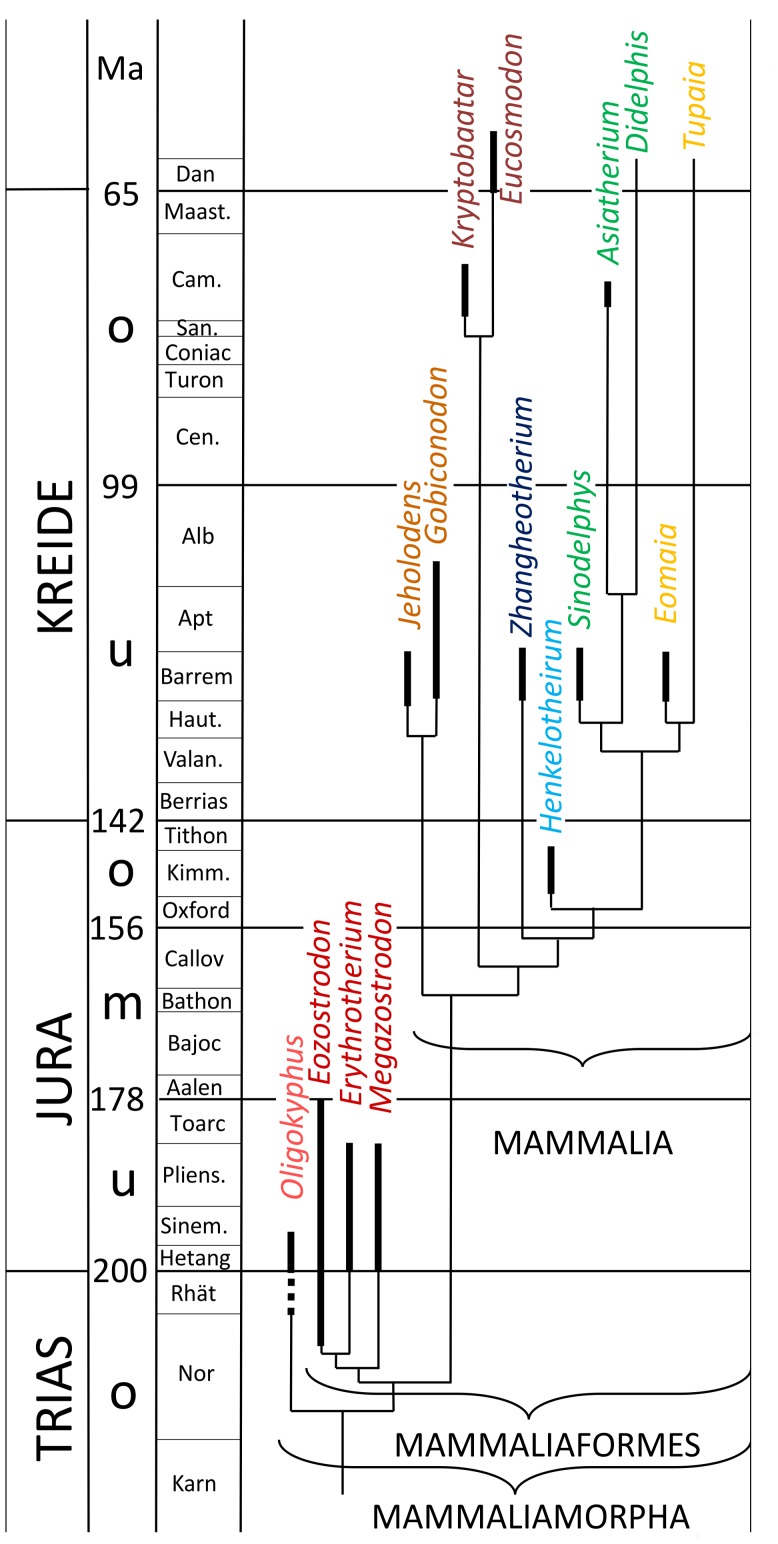
Cladogram of the fossil Mammaliamorpha mentioned in the text. The data were taken from [17], [20] and Fernando Abdala (pers. comm. 2012).

Two cladograms (Figs. 1 and 2) present the systematics of the Synapsida mentioned in the text. For brevity usually only the genus is mentioned in the text. When several specimens of one genus were measured and the observations were made on only one of those specimens, the museum identification number of the respective specimen is added.

### 2 Terminology

Wherever possible we refer to the anatomical nomenclature according to the latest standards of the Federative Committee on Anatomical Terminology (FCAT [21]). In a few cases it was necessary to use an amended nomenclature for a better understanding of the structures and biomechanics, especially for structures without an anatomical name.

**Figure 3:**
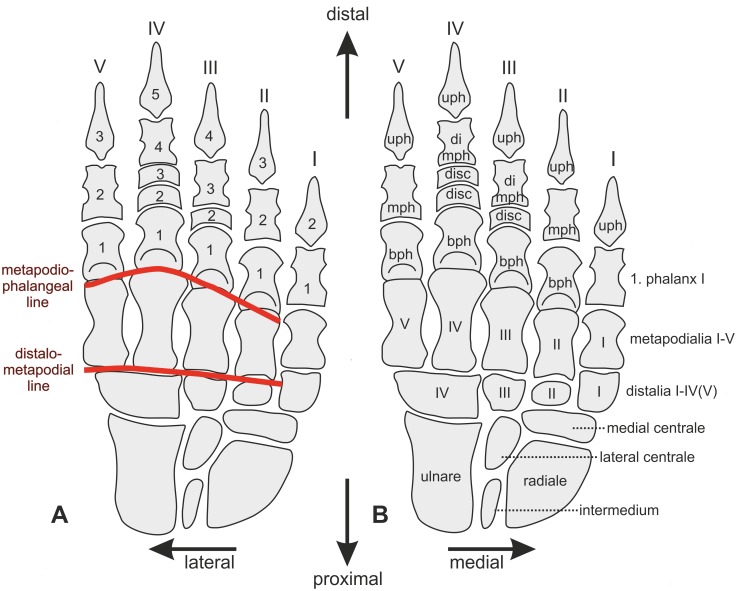
Nomenclature of manual bones in zero position (dorsal view) and articulation lines (in red). A: Nomenclature for phalanges according to their number per digit. B: Topographic nomenclature of phalanges, carpalia and metapodialia. Abbreviations: **bph** basal phalanx, **mph** middle phalanx, **di mph** distally situated middle phalanx, **disc** discoidal phalanx or proximally situated middle phalanx, **uph** ungual phalanx.

**Zero** and **standard positions:** The **zero position** of manus and pes is defined here as the position of the bones aligned in a straight, longitudinally orientated chain, without any longitudinal and transverse arch or digital arcade (Fig. 3). This mostly unnatural position is used to describe the position, orientation and the direction of movement of a bone. The **standard position** describes the position of the bones of manus and pes that likely occurred in the habitual posture of the living animal with a longitudinal and transverse arch, the spreading of the digits and, where present, a digital arcade.

**Phalanges and digital joints:** ungual or terminal joint = articulation between ungual phalanx and penultimate phalanx (digit I–V)

interphalangeal or middle joint = articulation between two phalanges excluding the terminal joint (digits II–V)

basal joint = articulation between phalanx and metapodiale (digits II–V)

metapodiophalangeal joint I = articulation between phalanx and metapodiale I (digit I)

distalometapodial joint I = articulation between metapodiale I and distale I (digit I)

centralodistal joint = articulations between the medial centrale and the distalia I and II (manus)

naviculodistal joint = articulations between the naviculare and the distalia I and II (pes)

The latter two joints form partially amphiarthroses with small excursion angles compared to the other joints.

Two different nomenclatures for the phalanges of digits II–V are used in the literature (Fig. 3). The phalanges are either numbered according to their order from proximal to distal, or they are named according to their topographical position as basal, middle and ungual [22]. In the latter case there may be more than one middle phalanx in digit III–V. The middle phalanges, which articulate with the ungual phalanges are also called “penultimate phalanges”. Both nomenclatures are used in this article, depending on the context.

**Articulation lines**: The distalometapodial line is the distally convex line passing through the distalometapodial joints of rays II–V. The metapodiophalangeal line is the distally convex line passing through the basal joints of digits II–V (Fig. 3).

**Figure 4:**
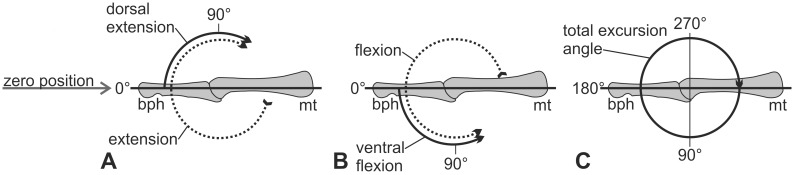
Nomenclature of excursion movements. A: Extension (dotted arrow) = the extension movement starting from maximum flexion; dorsal extension (solid arrow) = extension movement starting from the zero position dorsally. B: Flexion (dotted arrow) = flexion movement starting from maximum extension; ventral flexion (solid arrow) = movement from the zero position ventrally. In this paper the excursion angles of the digital joints are described in degrees of dorsal extension or ventral flexion, respectively. C: Total excursion angle = angle of increasing extension starting from the axis of the proximal segment of the joint. In the literature, the movement in a digital joint is often described in degrees of total excursion angle (e.g. [23]). Abbreviations: **bph** basal phalanx, **mt** metatarsale.

**Excursion angles/flexion/extension:** Extension is the movement of the distal phalanx in a digital joint in a dorsal direction, whereas flexion refers to the movement in palmar or plantar direction, respectively (Figs. 4A and B). The terms dorsal extension and ventral flexion describe an extension or flexion movement starting from the zero position, which is a reference position that makes the degree of flexion and extension comparable throughout all taxa. In this paper the excursion angle is always measured with respect to the zero position (Figs. 4A and B). It describes the actual mobility of the bone and is measured in degrees relative to the zero position. In the literature a total excursion angle is often given, which refers to the axis of the proximal segment of the digital joint and increases with increasing extension of the distal segment (Fig. 4C). In the zero position all autopodial elements are arranged in a line and thus the zero position equals 180° degrees of total excursion angle.

**Circumduction** is the rotation of a bone around a process, pivot or convexity of an adjacent bone.

**Opposition/reposition**: Following Napier [24], opposition is a combination of flexion, abduction and rotation. Other authors describe these movements as a combined flexion, adduction and rotation ([25], p. 448; [26]). Rauber and Kopsch [27] integrated both views and describe the opposition movement of the human thumb as follows: “Opposition starts by extension and abduction of the thumb; afterwards the thumb makes a combined movement, which is composed of flexion, adduction and inward rotation.” ([27], p. 440; translated from German).

These authors address primates in their definition, which possess nails and apical pads, so that the ventral face of the thumb is different than the dorsal one. For this reason, inward rotation of the thumb during opposition is essential in these forms. The same holds true for Synapsida bearing claws, because during gripping the claws are flexed and have to converge toward each other for a firm grip. This convergence can be brought about just by the general movement of digit I described by Rauber & Kopsch [27] for the human thumb.

**Pseudo-opposition** is the opposition of ray I brought about by a converging movement of the digital tips, abduction and flexion of the autopodial rays, but without a medial rotation ([24], p. 58, 82).

**Prehensility** is the efficiency of the converging movement of the digital tips. After Napier ([28], p. 116–117), an autopodium is prehensile when it has the ability to hold an item by its own. Animals with prehensile autopodia are, e.g., Old World primates, but also *Callithrix* with a pseudo-opposable ray I in the manus. Basal Marsupialia have a prehensile pes [24]. In fossil forms the type of prehensility cannot be positively verified. Therefore, the autopodia of fossil forms are described as prehensile if their digital tips can approach each other and if ray I is opposable or pseudo-opposable.

**The term digital arcade** refers to the ventral inclination of the distally situated phalanx against the proximally situated phalanx or metapodiale in a digital joint during most of the stride. At the end of the propulsion phase extension up to zero position may take place, but no dorsal extension. A digital arcade can occur in the terminal joint as in many reptiles, or in the middle joint of digits II–V as in most mammals, some geckos and turtles. It also occurs between phalanx and metapodiale in ray I of most mammals. The digits of mammals with a digital arcade cannot be dorsally extended in the middle joint of ray II–V and the metapodiophalangeal joint in ray I or only very slightly. The middle parts of their digits show no or only very weak contact with the substrate during the propulsion phase. The digital arcade in mammals is also referred to as autopodial pre-vault (“Vorgewölbe” [29]).

**Digital index** refers to the length of the middle phalanx, or in digits with discoidal phalanges of all middle phalanges, in comparison to the length of the corresponding basal phalanx of digits II–V.

**Autopodial rotation** is either lateral rotation of the autopodium on the substrate together with the rotation of the zeugopodium in animals with abducted limbs. Or it is the compensation of the rotation of the zeugopodium in the rolling mode of the autopodium. The latter takes place by abduction and rotation in the autopodial joints or by metapodial rotation (see below).

**Metapodial rotation**: Compensation of the rotation of the zeugopodium in animals with abducted limbs in the metapodium. The compensation takes place by rolling around an oblique axis through the heads of metapodialia II–IV of different lengths and/or by a higher ventral flexion of each metapodiale relative to the more medial metapodiale. The latter appears in the pes of *Caiman* [30].

### 3 Methods

#### The autopodia are described according to the zero position

Most of the fossil taxa and *Didelphis* were measured with calipers, with an accuracy of ±0.05 mm in the BP-fossils and ±0.02 mm in all others. Most Mesozoic Mammaliamorpha were measured with the aid of a stereo microscope, using calipers or, in case of *Morganucodon* and *Zhangheotherium* IVPP V7466, a reticle. *Henkelotherium* was photographed with a Zeiss Axiocam digital camera. Its bones were then measured on the photographs with the Zeiss Axio Vision 2.05 program. In most cases the maximum distances between the articular facets were measured, and not those of adjacent processes. For metapodiale I and basal and ungual phalanges, however, the distodorsal protrusion of metapodiale I and the basal phalanges and the proximodorsal process of the ungual phalanx were included in the measurement because in most cases the fossils were prepared in such a way that the joint facets were not exposed. Therefore, the bones were measured in dorsal aspect whenever possible.

The excursion angles of the first digit were measured by carefully moving the original bones of those fossils in which the bones were free of matrix *(Titanophoneus* PIN 157/1, *Biarmosuchus* PIN 1758/320, *Gorgonops torvus* BP/1/4089, *Theriognathus* NHMUK R 5694). In a few other specimens, where the bones were in articulation, the excursion angles were estimated as precisely as possible from the originals and/or photographs from different aspects. This method yielded approximations only. A micro-CT scanner and 3-D imaging would yield more precise results. However, the use of these tools was not practical for this comparative study, which included the investigation of many specimens in different museums.

As in extant tetrapods, the joint cartilage certainly did influence the excursion angle of the digital joints in the living animal to an unknown extent.

### 4 Reconstruction of the Standard Position

The standard position (see above) was reconstructed from intermediate positions of the excursion angles in the digital joints. Especially in the metapodiophalangeal joint I, an evident “preferred position” can be reconstructed based on an area with reduced curvature of the respective joint facets. In this position with a high joint contact the joint is stable, but still shows all degrees of freedom. The degree of flexion of the first phalanx was estimated and could thus be used to define the approximate standard position in most cases, because the distal part of phalanx 1 together with the flexor tubercle of the ungual phalanx, and very likely also the proximal part of metapodiale I, contacted the substrate via a digital pad or the sole pad.

Maximum flexion of all joints in ray I does not allow a normal stance, so that maximum flexion was excluded in the reconstruction of the standard position, whereas maximum extension occurs in the extreme position of the autopodium at the end of the propulsion phase.

## Osteology

### 1 Distale I of Manus and Pes

**Figure 5:**
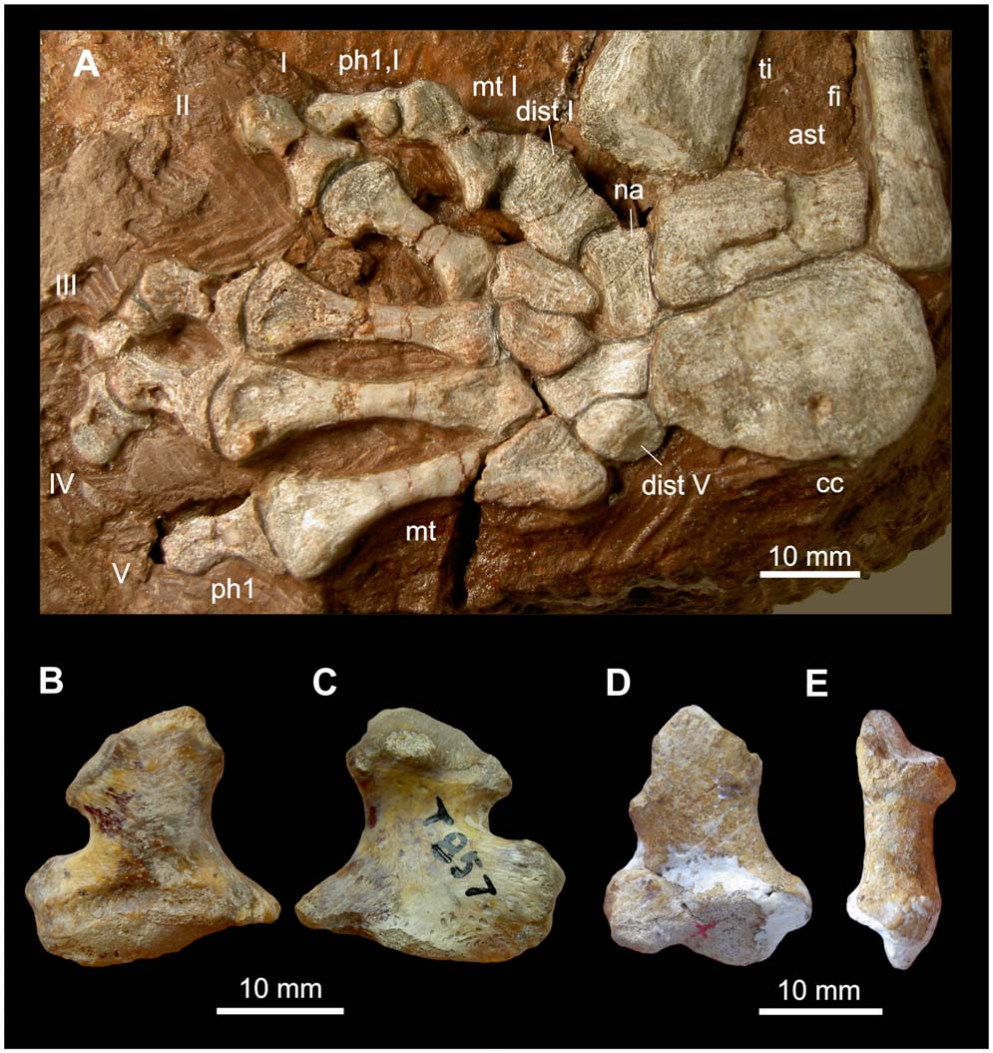
Ray I in *Dimetrodon* and *Biarmosuchus*. A: *Dimetrodon teutonis* MNG 10654: Left pes in dorsal aspect. B, C: *Dimetrodon* CAMZM T 875, right metapodiale (manus or pes?) in dorsal (B) and ventral aspect (C). D, E: *Biarmosuchus* PIN 1758/320, right metacarpale in dorsal (D) and lateral aspect (E). Abbreviations: **ast** astragalus, **cc** calcaneus, **dist** distale, **fi** fibula, **mt** metatarsale, **na** naviculare, **ph** phalanx, **ti** tibia.

In dorsal view, distale I is approximately quadratic or rectangular in outline, with a longitudinally directed long axis. In most Therapsida and *Dimetrodon*, distale I is connected with distale II and the medial centrale in the manus and with the naviculare in the pes, respectively, by amphiarthroses or articulation types with small excursion angles. At the distolateral face of distale I there is often also a small contact between distale I and metapodiale II. Topographically, distale I of Synapsida is mostly aligned with the row of the distalia II–V proximally and also with the row of metapodialia II–V distally. Therefore, distale I protrudes medial to metapodiale II and expands beyond the distally convex distalometapodial line connecting the distalometapodial joints II–V (e.g. Fig. 5A, Table 1). In some members of Synapsida, however, distale I is topographically restricted to the row of distalia. In the pes of *Ophiacodon* and possibly also in its manus, as well as in the manus of *Sinodelphys* and *Asiatherium*, the distalometapodial joint I is aligned in the distalometapodial line. Thus, distale I is situated within the row of the distalia II–V. In all other taxa investigated, there is a distal shift of distale I (Table 1), which is largest in non-kannemeyeriiform dicynodonts, where distale I may lie entirely distal to the distalometapodial line ([17], see further references in there).

**Table 1:** Topographical position of distale I and metapodiale I.

taxon		di I	mp I	taxon		di I	mp I
**PELYCOSAURIA**		**in %**				**in %**	
* Edaphosaurus* NHMUK R 9204	m	14	−	*Microgomphodon* SAM-PK-K10160	m	40	+
* Ophiacodon* AMNH 4776	m	0(?)	−	?Therocephalia TM 4696	m	30	0
* Edaphosaurus* NHMUK R 9204	p	21	0	*Glanosuchus* SAM-PK-12051	p	38	0
* Ophiacodon* AMNH 4781	p	0	−	*Theriognathus* BP/1/182	p	32	0
* Dimetrodon* MNG 10654	p	50	+	*Ictidosuchoides* SAM-PK-K10704	p	29	−
**BIARMOSUCHIA**				**CYNODONTIA**			
* Biarmosuchus* PIN 1758/320*	m	10	−	*Procynosuchus* TSK 34	m	33	
**DINOCEPHALIA**				*Procynosuchus* RC92	m	50	+
* Titanophoneus* PIN 157/1*	m	17	−	*Galesaurus* BP/1/4637	m	33	+
* Titanophoneus* PIN 157/1*	p	8	−	*Galesaurus* BP/1/2513	m	33	+
**GORGONOPSIA**				*Thrinaxodon* BP/1/2776	m	23	+
* Arctognathus* SAM-PK-3329	m	47		*Thrinaxodon* BP/1/1737	m	29	+
* Aelurognathus* SAM-PK-2342	m	34	+	*Diademodon* NHMUK R-3581	m	16	+
* cf. Rubidgea* BP/1/1210	m	30	+	*Chiniquodon* MCZ 3781	m	25	
gorgonopsian BP/1/4259	m	40	0	*Galesaurus* BP/1/4506	p	50	+
gorgonopsian SAM-PK-K4441	m	16	−	*Diademodon* NHMUK R-3581	p	36	+
* Rubidgea* BP/1/2167	p	32		*?Scalenodon* NHMUK R 9391	p	27	+
gorgonopsian SAM-PK-K4441	p	20	−	**MES MAMMALIAMORPHA**			
**THEROCEPHALIA**				*Jeholodens* GMV 2139a	m	12	−
* Glanosuchus* SAM-PK-K7809	m	12		*Zhangheotherium* IVPP V7466	m	6	−
* Glanosuchus* CGS RS 424	m	27	+	*Sinodelphys* CAGS00-IG03	m	0	−
* Theriognathus* NHMUK R 5694	m	27	0	*Asiatherium* PIRAS 3907	m	0	−
* Ictidosuchoides* BP/1/4092	m	25		*Megazostrodon* NHMUK M 26407	p	21	−
* Ictidosuchoides* CGS CM86–655	m	23	−	*Jeholodens* GMV 2139a	p	10	−
* Regisaurus* BP/1/3973	m	30	−	*Kryptobaatar* ZPAL MgM-I/41	p	21	−
* Aelurosuchus* SAM-PK-5875	m	20	0	*Eomaia* CAGS01-IG-1a	p	18	−

Proxies of the portion of distale I (**di I**) that lies medial to metapodiale II in those autopodia, which are basically preserved in articulation, calculated in per cent of the length of metapodiale II. Distal end of metapodiale I (**mp I**) compared to metapodiale II in autopodia basically preserved in articulation: The distal end of metapodiale I extends distally to metapodiale II (+), terminates proximally to it (−) or lies level with it (0). Abbreviations: **di** distale, **Mes** Mesozoic, **mp** metapodiale.

*Titanophoneus PIN 157/1 after Orlov ([1] Figs. 47 and 54), *Biarmosuchus* PIN 1758/320 after Chudinov ([31] Fig. 1).

In the row of distalia and the proximal ends of metapodialia, the synapsid autopodium shows a transverse arch (Table 2). As is seen in Table 2, the transverse arch is less pronounced in the phylogenetically more basal forms and more pronounced in the more derived forms of Synapsida.

**Table 2:** Transverse arches of synapsid autopodia.

taxa		section of measurement	preservation state	I againstIII	comment
**BIARMOSUCHIA**		****	****		
* Biarmosuchus* PIN 1758/320	m	metacarpalia pr	no matrix	20°	
**DINOCEPHALIA**					
* Anteosaurus* SAM-PK-4323	m	metacarpalia pr	pr asp	20°	
* Titanophoneus* PIN 157/1	m	mc pr, distalia	no matrix	20°	
**GORGONOPSIA**					
gorgonopsian BP/1/4259	m	metacarpalia pr	*in situ*	35°	
**THEROCEPHALIA**					
* Glanosuchus* SAM-PK-12051	m	metacarpalia pr	di asp	30°	
* Glanosuchus* CGS RS424	m	distalia	*in situ*	30°	
* Theriognathus* NHMUK R 5694*	m	distalia	*in situ*	30°	cast
* Ictidosuchoides* CGS CM86–655	m	distalia	*in situ*	35°	
**CYNODONTIA**					
* Procynosuchus* RC92	m	distalia	*in situ*	45°	
* Thrinaxodon* BP/1/5558	m	distalia	*in situ*	35°	
* Diademodon* NHMUK R-3581	m	distalia	*in situ*	35°	
* ?Scalenodon* NHMUK R 9391	p	metatarsalia pr	*in situ*	35°	
* Asiatherium* PIRAS 3907	m	distalia	*in situ*	45°	cast

The values are proxies for the angle between the dorsal faces of distalia I and III or of the proximal parts of metapodialia I and III (measurements taken from photos). The values could only be measured in those few specimens in which the row of distalia or the proximal ends of metapodialia (**mc pr**) are exposed in transverse section or the transverse arch is preserved *in situ*. In the latter case the values do not necessarily represent the maximum possible arch, so these values may be too low in some specimens. Abbreviations: **asp** aspect, **di** distal, **m** manus, **mc** metacarpalia, **p** pes, **pr** proximal.

*Theriognathus NHMUK R 5694 cast of the manus, which represents the articulated state, in which the manus was found (Sandra Chapman, pers. comm. 2012).

### 2 Metapodiale I of Manus and Pes

In non-mammaliamorph Therapsida and *Dimetrodon*, metapodiale I has a completely different morphology than metapodialia II–V: It strikingly resembles a basal phalanx in form and function (e.g. Fig. 5A). This is not only seen in the distal joint of metapodiale I (see below), but also in its topography, so that in the pes of *Dimetrodon* and in non-mammaliamorph Cynodontia, metapodiale I protrudes distally beyond metapodiale II (Table 1). In the other therapsid groups, the distal end of the bone terminates distal to or level with metapodiale II, and in some cases also proximal to it. In all Mesozoic Mammaliamorpha, however, it terminates proximal to the distal end of metapodiale II (Table 1).

Compared to the basal phalanges II–V, metapodiale I is broader at its base. In some taxa of non-therapsid Eupelycosauria and early Therapsida, a big, rugose process arises from the medial face of the base of metapodiale I, which served for the insertion of abductor, flexor and extensor tendons [1], [32]. This process is particularly large in *Titanophoneus* and *Dimetrodon* (e.g. Figs. 5B and C). In *Titanophoneus*, the process reaches half the length of metapodiale I. The width of the process is approximately 17% of the basal width of metapodiale I in the manus and about 19% in the pes. In metapodiale I of *Dimetrodon sp.* CAMZM T 857 it measures about 28% of its basal width (an assignment to manus or pes is not possible).

### 3 First Phalanx I of Manus and Pes

The first phalanx I in Therapsida including Mesozoic Mammaliamorpha resembles the penultimate phalanges II–V in the shape of the articular head (see below), the shape of the shaft and in the proximal width.

### 4 Ratio First Phalanx I to Metapodiale I

The ratio of the length of the middle phalanges (mph) to the length of the corresponding basal phalanges (bph) in digits II–V, the digital index, shows roughly the same value in digits II–V of the same autopodium in Therapsida [17]. Regarding ray I, the ratio of the length of the first phalanx I (ph 1 I) to that of metapodiale I (mp I) in most forms roughly equals the digital index of the lateral digits II–V. The difference of the index in autopodial ray I (L_ph 1 I_ 100/L_mp I_) to the index in autopodial ray II (L_mph_ 100/L_bph_) usually is below 20%. Taxa in which this difference is greater than 20% are listed in Table 3. In *cf. Rubidgea* and *Bauria* the index of autopodial ray I (L_ph__1 I_ 100/L_mp I_) is lower than the digital index in digit II (L_mph_ 100/L_bph_). In *Asiatherium* the index of ray I is higher than the digital index of digit II. The other differences may be due to measuring artefacts (see comments in Table 3).

**Table 3:** Ratio between the length of the first phalanx I and length of the metapodiale I (in %) compared with the length ratios between middle phalanx II and basal phalanx II.

taxon		ratio		difference		comment
		1st phI/mpI	mph/bph II	value of index 1		
		×100	×100	minus		
		index 1	index 2	value of index 2		
GORGONOPSIA						
* cf. Rubidgea* BP/1/1210	m	80	107	−27		
gorgonopsian BP/1/4259	m	102	80		22	II 2 fractured
**THEROCEPHALIA**						
therocephalian BP/1/5898	m	106	75		31	mc I (?); palmar measurement
* Bauria* CAMZM T 373	p	74	104	−30		cast of AMNH 5622; II 2 compressed
Therocephalian NMQR 3530	p	65	99	−34		
**CYNODONTIA**						
* Asiatherium* PIRAS 3907	m	84	53		31	cast

Only taxa, in which the difference between the indices in autopodial rays I and II is more than 20% are listed. Abbreviations: **bph** basal phalanx, **m** manus, **mc** metacarpale, **mph** middle phalanx, **mp** metapodiale, **p** pes, **ph** phalanx.

## Arthrology

In this study we do not follow the phylogeny of the genera as represented in Fig. 1 and 2 to the very detail. The preservation state of ray I of the respective fossil is more important for the order we use here. Thus, in each evolutionary level we describe the best preserved and best exposed ray I first, followed by the less well preserved or exposed ones, always with reference to the best description.

### 1 Distalometapodial Joint I of Manus and Pes

#### Dinocephalian *Titanophoneus* PIN 157/1

**Figure 6:**
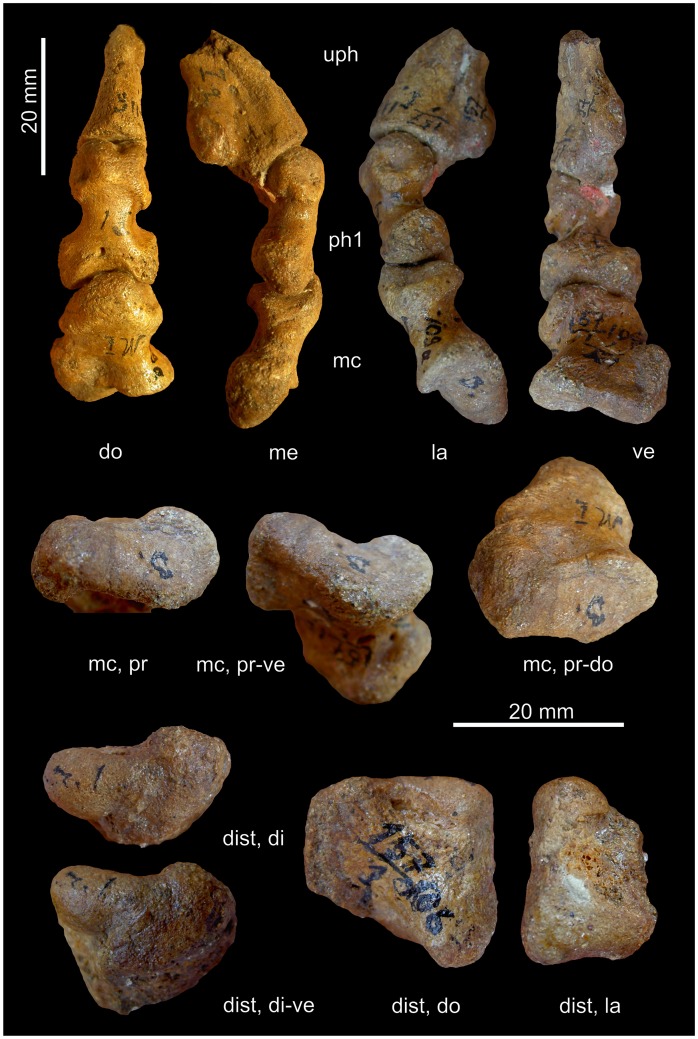
Ray I of *Titanophoneus* PIN 157/1, right manus. Abbreviations: **di** distal, **dist** distale, **do** dorsal, **la** lateral, **mc** metacarpale, **me** medial, **pr** proximal, **ve** ventral.

**Figure 7:**
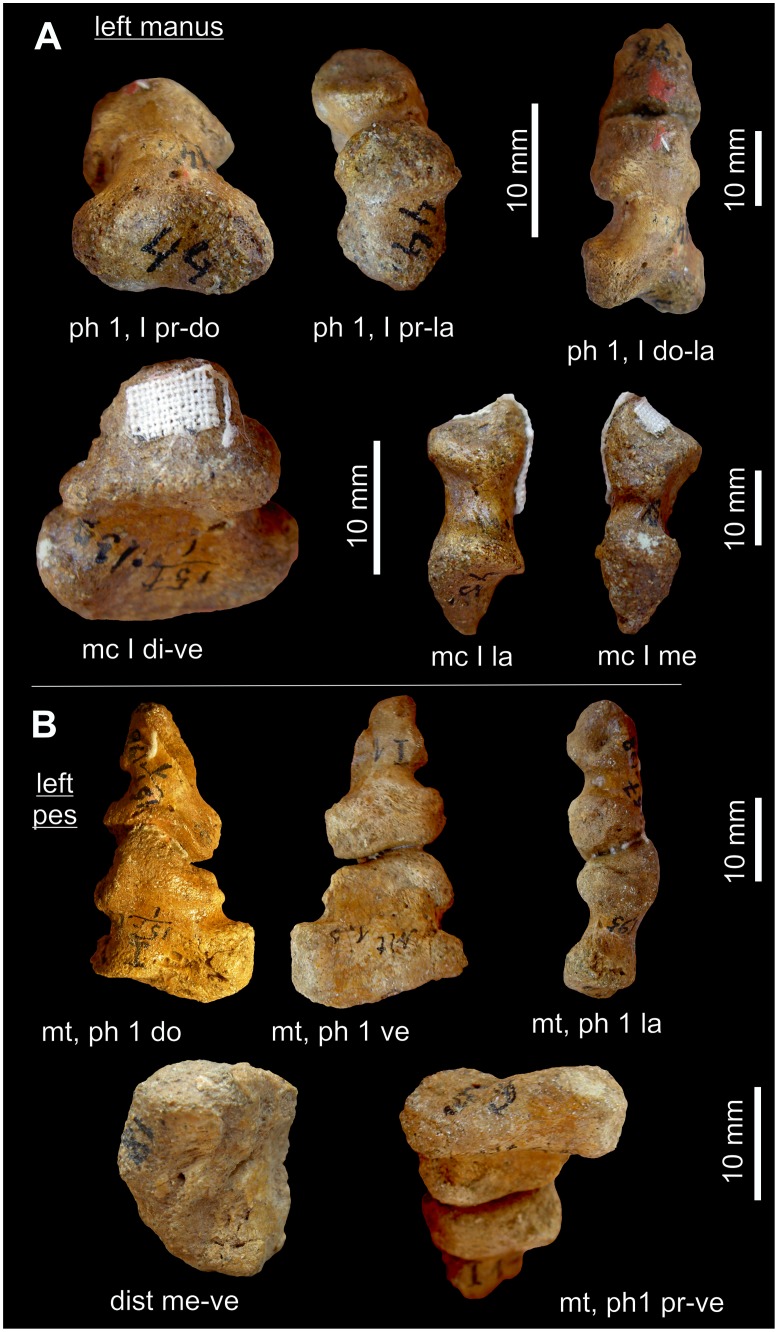
Ray I of *Titanophoneus* PIN 157/1. A: Metapodiophalangeal joint I of the left manus. B: Ray I of the left pes. Abbreviations: **di** distal, **dist** distale, **do** dorsal, **la** lateral, **mc** metacarpale, **me** medial, **mt** metatarsale, **ph** phalanx, **pr** proximal, **ve** ventral.

The distalometapodial joint I is well preserved in the right manus. It is also well preserved in the proximal part of metatarsale I in the left pes, but the surfaces of distale I in the left pes are partly eroded, as is the surface of distale I in the left manus (Figs. 6 and 7).

The distalometapodial joint I is an asymmetrical saddle joint. The proximal articular facet of metacarpale I in the right manus shows a concavity that runs from the central part of the dorsal border in ventrolateral direction. At the ventrolateral corner the concavity terminates in a sharp ridge, which borders the facet on the lateroventral side. The medial convexity is very large in relation to the entire facet and exhibits a convex articular face tapering conically from the medial face to the middle portion of the ventral border of the proximal articular facet. The dorsolateral border of the facet forms a sharp ridge angled at 70° against the shaft. The above mentioned concavity running from the dorsal to the ventrolateral side widens in its lateral and lateroventral part of the proximal articular facet, so that in this part the joint forms an ellipsoidal socket (Fig. 6).

The distal joint facet of distale I (Fig. 6) bears an ellipsoidal condyle on its lateral face with its long axis being orientated lateromedially. On the proximoventral and proximodorsal border of the condyle are two sulci whose surfaces bear rugosities and pits for the insertion of a strong articular capsule. A convexity occupies the dorsomedial part of the facet. The ventrodistally facing facet of this convexity shows an extension in the ventroproximal direction, curving slightly laterally. This extension itself is slightly convex. The ventroproximal extension of the dorsomedial convexity and the lateral condyle are separated by a faint concavity.

The asymmetric saddle joints of manus and pes are very similar to each other. However, there are slight differences between them: The joint in the pes shows less relief (Figs. 6 and 7), thus, the socket of metatarsale I and the condyle of pedal distale I are less pronounced. However, the medial convexity of metatarsale I is very strong and extends further laterally than in the manus.

The lateral part of the concavity of metapodiale I, which resembles an elliptical socket, articulated with the lateral condyle of distale I in the living animal. Metapodiale I could be circumducted in a medial direction around the lateral condyle of distale I. During that movement, metapodiale I additionally rotated around the transverse long axis of the medial convexity of metapodiale I, causing the flexion of the digit. There was a tight articular contact during flexion and circumduction. The maximum possible rotation of metacarpale I in that joint was 45° (Table 4).

**Table 4:** Proxies of the dorsomedial and ventral convexities or margins of distale I and the excursion angles in the distalometapodial joint I.

taxon		A	B	C	D	E	F	G
**EUPELYCOSAURIA**		**%**	**%**					
* Dimetrodon* CAMZM T 857	*					min	yes	no
* Dimetrodon* MNG 10654	p	22						
**BIARMOSUCHIA**								
* Biarmosuchus* PIN 1758/320	m			50°			yes	no
**DINOCEPHALIA**								
* Titanophoneus* PIN 157/1	m	3	0	50°		min	45°	no
**GORGONOPSIA**								
* cf. Rubidgea* BP/1/1210	m	25–26					>35°	
gorgonopsian BP/1/4259	m	25–26	27					
gorgonopsian CGS CM 86–471	m	25–26	18					
gorgonopsian BP/1/600	m		18				>20°	
gorgonopsian SAM-PK-K4441	m	25						
* Gorgonops* BP/1/4089	p	15	6	60°	45°	20°	yes	yes
gorgonopsian SAM-PK-K4441	p	10	13					
**THEROCEPHALIA**								
* Glanosuchus* CGS RS424	m	17						
* Theriognathus* NHMUK R 5694	m		13		10°	20°	20°	20°
* Ictidosuchoides* CGS-CM86–655	m	7						
* Glanosuchus* SAM-PK-12051	p	24						
**CYNODONTIA**								
* Procynosuchus* RC 92	m	14						
* Procynosuchus* TSK 34	m	17						
* Galesaurus* SAM-PK-K10468	m		5					
* Galesaurus* BP/1/2513a	m	14						
* Thrinaxodon* BP/1/2776	m		20					
* Diademodon* NHMUK R 3581	m	0						
* Chiniquodon* PVL 3820 (photo)	m		19					
* Galesaurus* BP/1/4506	p	6						
* Diademodon* NHMUK R 3581	p	7						

A: Length of dorsomedial or dorsal convexity or elongated margin of distale I. B: Length of ventral or ventrolateral convexity of distale I. C–G: Excursion angles: C: dorsal extension, D: ventral flexion, E: medial abduction, F: medial circumduction, G: lateral circumduction. Abbreviations: **m** manus, **min** minimal, **p** pes.

*The assignment to manus or pes is not possible.

In the maximum dorsal extension of about 50° with respect to the zero position, the dorsolateral ridge of metapodiale I interlocks with the sulcus on the proximodorsal end of the lateral condyle of distale I. During dorsal extension the joint contact decreases and therefore is less stable in the extended than in the flexed position. In this joint, only very slight abduction was possible.

#### Non-therapsid Eupelycosauria

In *Dimetrodon* CAMZM T 857 (manus or pes; Figs. 5B and C) and in *Dimetrodon* AMNH 24810 (manus or pes) the proximal articular facet of metapodiale I is visible. In the manus and pes of *Ophiacodon* AMNH 4781 the distalometapodial joint I is only visible in dorsal view.

The distalometapodial joint I of *Dimetrodon* is similar to that of *Titanophoneus*. The concavity of metapodiale I runs from the dorsal side in a ventrolateral direction, but is not as pronounced as in *Titanophoneus*. In the proximal articular facet of metapodiale I, the dorsolateral ridge has a length of 44% of the total basal width of the bone, exactly as in *Titanophoneus.* However, in *Dimetrodon* it continues to the lateral side. It encircles the wide lateral part of the concavity dorsally and laterally. On the ventral side of metapodiale I the medial convexity continues into a depression with a rugose surface for the insertion of a tendon or the articular capsule. On the proximodorsal border of the joint facet, there is also a sulcus for insertion of the strong articular capsule. In the pes of *Dimetrodon teutonis* MNG 10654 (Fig. 5A) the length of the dorsomedial convexity on the distal face of distale I is about 22% of the total length of distale I. With respect to this convexity, *Dimetrodon* resembles more the gorgonopsian condition than that of *Titanophoneus* (Table 4).

In *Dimetrodon* the mobility of the joint must have been similar to that of *Titanophoneus*, but the excursion angles might have been slightly smaller.

In the manus and pes of *Ophiacodon* AMNH 4781, the distalometapodial joint I is a saddle joint, but only shows a faint relief. Apparently, the manus and pes of *Ophiacodon* had limited mobility in that joint.

#### Biarmosuchia

In the manus of *Biarmosuchus* PIN 1758/320, the joint facets of the distalometapodial joint I are exposed, but the joint surfaces are eroded (Figs. 5D and E). In the pes of *Hipposaurus* SAM-PK-8950 the joint is seen from the ventral side.

The distalometapodial joint I in Biarmosuchia is of the same type as in *Titanophoneus*. In metacarpale I of *Biarmosuchus* the socket-like concavity on the lateral face of the joint facet is larger in relation to the entire facet than in *Titanophoneus*, showing a prominent ventral ridge, but the saddle joint character as a whole is less evident than in *Titanophoneus*. However, this may be due to the bad preservation of the bone. In the pes of *Hipposaurus* the lateral condyle of distale I is wide in relation to the width of the bone and is pronounced.

The well-developed socket-like structure of the concavity on metacarpale I of *Biarmosuchus* with its prominent ventral ridge, and the pronounced condyle on distale I of *Hipposaurus* suggest that circumduction of metapodiale I in the medial direction had a large excursion angle in both species. Dorsal extension up to 50° was possible in *Biarmosuchus*, with respect to the zero position (Table 4).

#### Gorgonopsia

**Figure 8:**
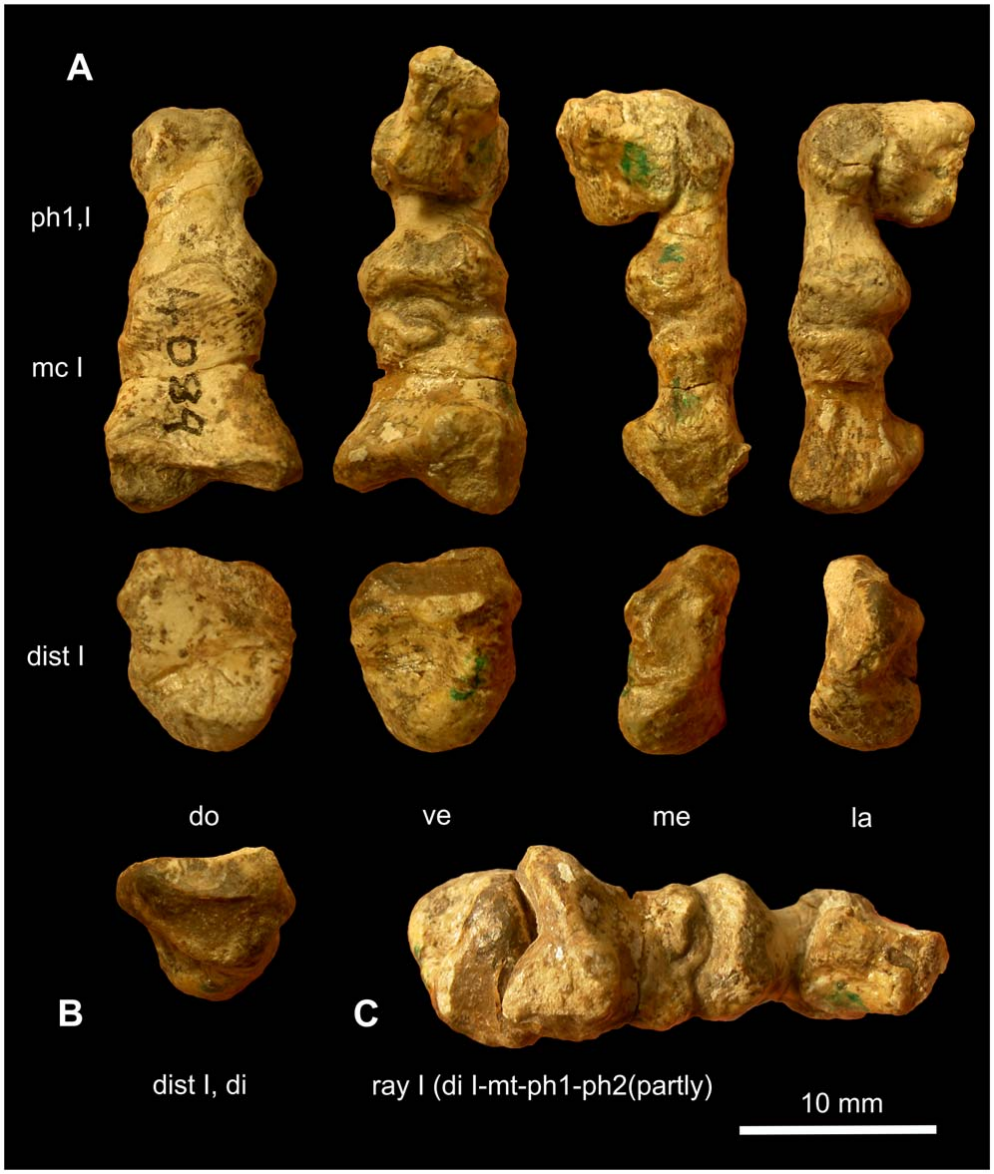
*Gorgonops* BP/1/4089: First ray of the right pes. A: Ray I in dorsal, ventral, medial and lateral view; B: Distale I in distal aspect; C: Ray I (di I-mt-ph1-ph2 (partly)) articulated as fossilized, mainly seen in plantar aspect, but hyperextended in the distalometapodial joint. Abbreviations: **di** distal, **dist** distale, **do** dorsal, **mc** metacarpale, **ph** phalanx, **ve** ventral.

**Figure 9:**
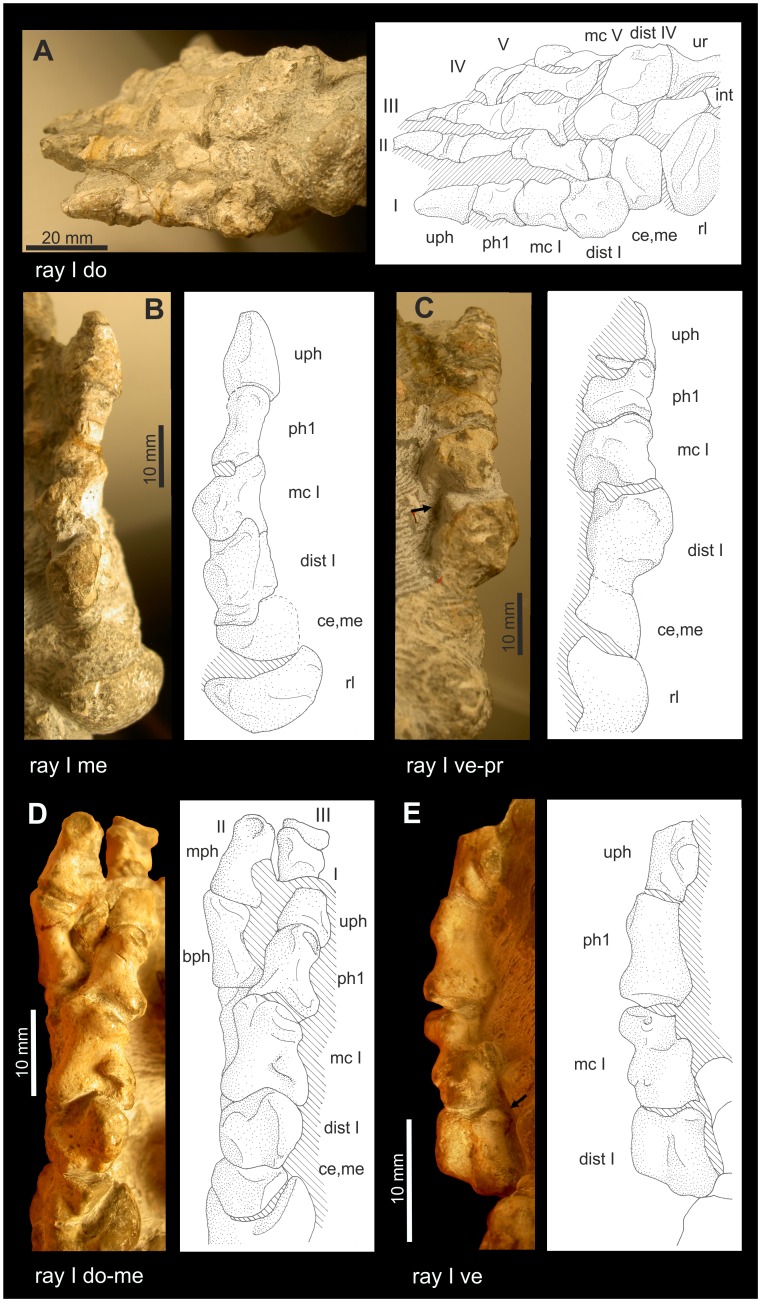
Pollex of Gorgonopsia. A–C: Gorgonopsian BP/1/4259: Right manus. First ray is seen in dorsal (A), medial (B) and ventroproximal (C) view. D−E: Gorgonopsian BP/1/600: Left manus. First ray represented in dorsomedial (D) and ventral (E) views. The arrow marks the ventral convexity of the distal face of distale I. Abbreviations: **ce** centrale, **dist** distale, **do** dorsal, **int** intermedium, **mc** metacarpale, **me** medial, **ph** phalanx, **pr** proximal, **rl** radiale, **uph** ungual phalanx, **ur** ulnare, **ve** ventral.

**Figure 10:**
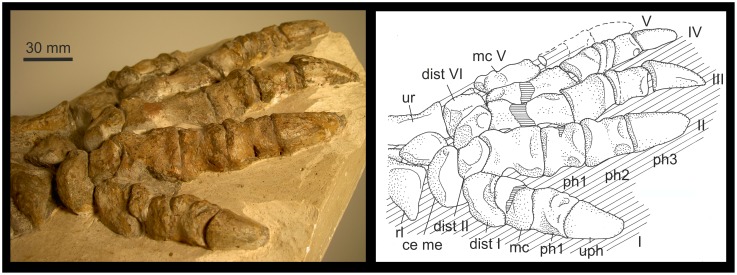
*Cf. Rubidgea* BP/1/1210: Ray I of the left manus. Ray I is seen in distodorsal aspect. Abbreviations: **ce** centrale, **dist** distale, **mc** metacarpale, **me** medial, **ph** phalanx, **rl** radiale, **uph** ungual phalanx, **ur** ulnare.

**General description**: The distalometapodial joint I of Gorgonopsia is saddle-shaped and resembles that of *Titanophoneus* and *Dimetrodon*, but is more symmetrical than in the latter. The joint facet of distale I is triangular in distal aspect (Fig. 8B), with a convexity present on the ventral or slightly lateroventral border of the facet (black arrows in Figs. 9C and E). Dorsal to the convexity is a shallow, approximately lateromedially oriented concavity. Except for gorgonopsian SAM-PK-K4441 (see below), the distodorsal part of distale I shows a transversally orientated lip bordering the facet dorsally (Fig. 8). The lip is bigger and distoproximally longer on the dorsomedial side than dorsolaterally, forming a convexity around which metapodiale I could be circumducted to some degree (Figs. 9A, B, D and 10). The dorsomedial convexity is distoproximally longer in Gorgonopsia when compared with that of *Titanophoneus*. On the dorsolateral side of distale I the lip flattens and continues in a convex ridge (Figs. 8B and C). This ridge might be homologous to the lateral condyle of distale I in *Titanophoneus*.

The ventrally situated convexity of the distal facet of distale I in Theriodontia varies in its distal extension, shape and position. It is sometimes nearly flat, like in the pes of *Gorgonops* BP/1/4089, and sometimes very prominent, like in the manus of the gorgonopsian BP/1/4259 (Table 4). The variation in the relief of the joint can either be due to a different morphology of the joint in different species, differences in manus and pes, or different ontogenetic stages or preservation states of the fossils. In juveniles the cartilaginous joint facets are not preserved.

On the ventral side of distale I, a ridge extends from the distoventral convexity, or the margin of the facet adjacent to this convexity, in a proximal direction. This ridge separates two indentations, which probably served as insertion pits for strong tendons of the joint capsule (Figs. 8A and 9E).

The concavity of metapodiale I is orientated mainly dorsoventrally, but at the same time extends to the dorsolateral part of the facet, which is connected to the convex dorsolateral situated ridge of distale I (Figs. 8A, C, 9A and 10). The dorsomedial part of the facet also bears a depression, which fits to the corresponding, dorsomedial convexity of distale I. Ventroproximally, the concavity separates the lateroventrally and medioventrally situated convexities. The convexities are subspherical and prominent; the medioventral convexity is slightly more prominent than the lateroventral one, both primarily directed in a ventral direction (Fig. 8C).

The medioventral and lateroventral convexity of metapodiale I are the part of the proximal portion of ray I where the load was transferred to the ground. The concavity between these convexities was the guiding sulcus for the flexor tendons. Especially on the medial and ventral side of the medioventral convexity, the margin of the articular facet often shows rugosities and sometimes small tubercles for the insertion of strong tendons and the articular capsule. Obviously the convexities of metapodiale I were a multifunctional structure in having been part of a saddle joint, transferring loads from the autopodium to the ground, and serving as strong insertion areas distally and medially for tendons and the joint capsule.

**Pes of** ***Gorgonops*** **BP/1/4089**: The joint facets of the distalometapodial joint I are free of matrix (Fig. 8). Therefore, the mobility of metapodiale I against distale I was directly tested by careful movement of the bones in the joint. However, the dorsomedial convexity of distale I is broken and fixed to metapodiale I. The entire facet of distale I seems to be slightly compressed dorsoventrally, and the surface of the facet of metapodiale I is incomplete. Because of this, the reconstruction of the excursion angles can only be approximated.

The relief of this joint is less pronounced than in that of the manus in other Gorgonopsia. The dorsomedial convexity of distale I has a distoproximal extension of about 15% of the dorsal length of distale I (Table 4). The dorsomedial convexity continues as a small convex ridge to the dorsolateral margin of the facet. The shallow concavity of distale I is directed more or less lateromedially (Fig. 8B). Ventrally, the triangular facet is bordered by a rim situated at the central part of the proximoventral face. Its distal part was probably cartilaginous, because the bone surface is missing at the tip of the margin, so it probably formed a convexity in the living animal. The margin protrudes mainly in a ventral direction and only slightly distally for about 6% of the ventral length of distale I (Table 4).

The concavity of metatarsale I extends from the ventral part of the joint in a dorsal direction. There is a slight extension of it onto the dorsolateral side of the respective metatarsale (Fig. 8A). The medioventral convexity of metatarsale I is ventrally more prominent than the lateroventral one and shows rugosities and tuberosities along the ventral and medial part of its distal margin.

A dorsal extension of metatarsale I about 60° might have been possible. On the ventral side, between the two convexities of metatarsale I is a small, transversely orientated ridge, which forms a bone lock when metatarsale I is flexed ventrally at an angle of about 45°. Abduction of metatarsale I of about 20° in a medial direction was possible.

During dorsal extension metatarsale I could be circumducted around the dorsomedial convexity of distale I, mainly in a medial direction. The lateral part of metatarsale I could move on the convex surface of the dorsolateral ridge of distale I during extension, but also in the concavity ventral to it during flexion. Therefore, unlike in *Titanophoneus*, some circumduction of metapodiale I could also take place in lateral direction (Table 4). Circumduction during flexion in a medial direction was probably also possible, but could not be tested due to the compressed state of the joint.

#### Manus of the gorgonopsians BP/1/4259, BP/1/600, CGS CM 86-471 and *cf*

***Rubidgea*** **BP/1/1210**: The manus in each of these fossils is preserved in articulation (Figs. 9 and 10). The distalometapodial joint I is visible in dorsal and ventral view. In *cf. Rubidgea* BP/1/1210 it is only seen in dorsal view.

The distoproximal extension of the dorsomedial convexity of distale I is about 25–26% the length of distale I in BP/1/4259, CGS CM 86–471 and *cf. Rubidgea* BP/1/1210 (Table 4). The ventral convexity of distale I, which lies slightly lateral to the centre of the ventral face of the bone, ranges between 18 and 27% of the entire ventral length of distale I (CGS CM 86–471 and BP/1/600 about 18%, BP/1/4259 about 27%). It was probably longer in the living animal, because in BP/1/600 and BP/1/4259 the surfaces on the convexities are lacking and the underlying porous structure suggests a cartilaginous cover of that area. In CGS CM 86–471 the distal part of the ventral convexity is slightly damaged.

The medioventral convexity of metacarpale I is very long in BP/1/4259 with respect to the length of metacarpale I and it is dorsoventrally high in BP/1/600 with respect to the height of the distal part of the bone. In BP/1/600 the distal margin of the medioventral convexity shows tubercles and rugosities for the insertion of the joint capsule or tendons. The pronounced rugosities suggest that the joint capsule must have been very massive and that it stabilized especially the medial part of the joint during flexion, extension and circumduction. In CGS CM 86–471, both convexities are nearly of the same size and show only small tuberosities, in particular on their medial side.

All four gorgonopsian manus, described in the last three paragraphs, are fossilized in articulation. In all of them, metapodiale I is circumducted in a medial direction around the dorsomedial convexity of distale I, so that the lateral part of the metapodiale I remained in close contact with the top of the dorsolateral ridge of distale I. The dorsoproximal border on the lateral side of metapodiale I is very sharp. Therefore, the dorsolateral part of the metacarpal facet in the manus must show a bigger concavity than in the pes of *Gorgonops* BP/1/4089.

In these four manus the range of circumduction of metapodiale I must have been larger than in the pes of *Gorgonops* BP/1/4089 due to the more pronounced relief of the respective joint surfaces. Especially, the circumduction in a medial direction around the dorsomedial and the ventral, respectively ventrolateral convexity of distale I was possible to a high degree. In *cf. Rubidgea* BP/1/1210, metapodiale I is preserved in a medially rotated position of about 35° against distale I. A lateral circumduction was also possible, but to a lesser degree.

**Manus and pes of the gorgonopsian SAM-PK-K4441**: The distalometapodial joint I is visible dorsally in both manus, but is partly damaged in the right. In the pes this joint is seen in dorsal and ventral views.

In SAM-PK-K4441 the dorsal convexity of distale I protrudes distally in the central part of the dorsal margin. Therefore, in contrast to the Gorgonopsia described above, there is only a central convexity, and the dorsal ridge is missing. The distoproximal extension of the central convexity in the left manus is about 25% of the total dorsal length of distale I (Table 4). However, distale I is very short with respect to the width of the joint. Thus, the convexity had probably only half the length of that seen in the manus of the other Gorgonopsia. In the pes it only shows a length of about 10% of the dorsal length of distale I, whereas the ventrolateral convexity in the pes is about 13% the ventral length of the bone.

The concavity of metapodiale I is very deep in the dorsoproximal part of metacarpale I. It is less pronounced in the pes. Metatarsale I is seen in ventral view, too, where the concavity of the proximal part terminates on the ventrolateral face.

Circumduction of metapodiale I around the dorsal convexity of distale I could take place in both medial as well as in lateral directions. Especially in the pes, movement in the medial direction was dominant, whereas movement in a lateral direction was restricted. During flexion an inevitable circumduction of metapodiale I in the medial direction occurred, which was supported by the ventrolateral convexity of distale I.

**Mobility**: As is concluded from the morphology of the distalometapodial joint I of the gorgonopsian SAM-PK-K4441 in dorsal view, the mobility in manus and pes is similar. Due to the lower relief of the joint surfaces, the excursion angles of metapodiale I are smaller in the pes than those of the manus. In the other fossils described here, the manus and pes are not preserved together, hence the same pattern is visible: The pes of *Gorgonops* BP/1/4089 shows a similar mobility as the described manus of the gorgonopsians BP/1/4259, BP/1/600, CGS CM 86–471 and *cf. Rubidgea* BP/1/1210, only the relief is less pronounced in the pes.

Abduction and adduction, flexion, extension, and rotation of metapodiale I were possible in the distalometapodial joint I. The convexities of distale I are more pronounced in Gorgonopsia than in most of other Therapsida. The dorsomedial convexity of the distal articular facet of distale I serves as a cone for circumduction of metapodiale I. This is also true for the ventrolateral convexity. In animals with a pronounced relief of this articular facet, a medially directed circumduction of more than 35° was possible. In contrast to *Titanophoneus* and *Dimetrodon*, some circumduction in a lateral direction was also possible. Because of the pronounced relief of the distalometapodial joint I in Gorgonopsia, the ability to abduct metapodiale I was better developed than in most other Therapsida.

#### Therocephalia

**Figure 11:**
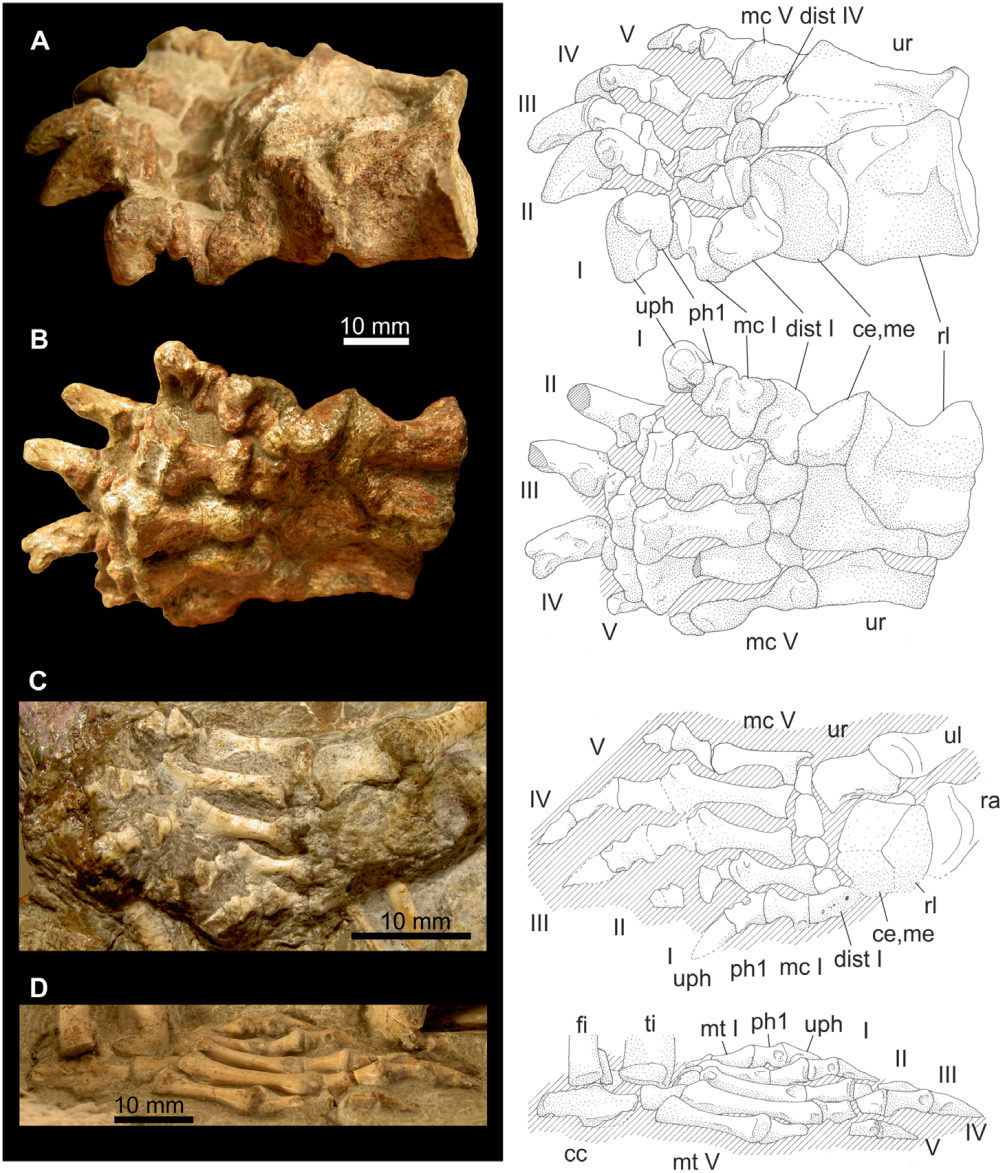
First ray in Therocephalia. A and B: *Glanosuchus* CGS RS424 right manus in dorsomedial (A) and ventromedial aspects (B). C: *Ictidosuchoides* CGS CM86–655: Right manus in dorsal view. D: *Ictidosuchoides* SAM-PK-K10704, right pes in dorsolateral view. Abbreviations: **cc** calcaneus, **ce** centrale, **dist** distale, **fi** fibula, **mc** metacarpale, **me** medial, **mt** metatarsale, **ph** phalanx, **ra** radius, **rl** radiale, **ti** tibia, **ur** ulnare, **uph** ungual phalanx.

**Figure 12:**
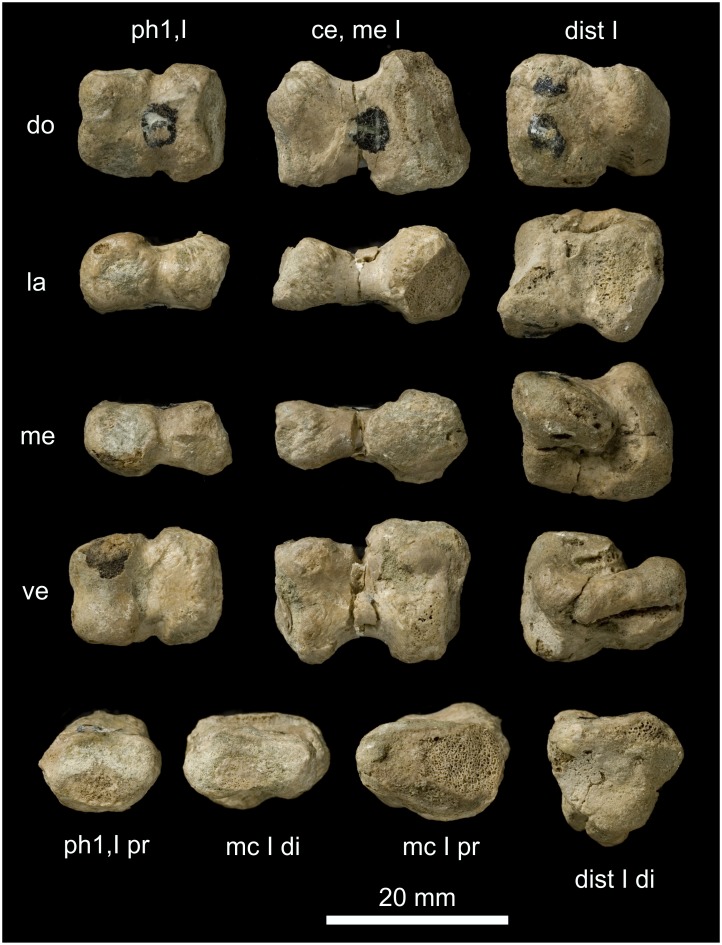
*Theriognathus* NHMUK R 5694, ray I of the right manus . Photos courtesy of NHMUK. Abbreviations: **ce** centrale, **di** distal, **dist** distale, **do** dorsal, **la** lateral, **mc** metacarpale, **me** medial, **ph** phalanx, **pr** proximal, **ve** ventral.

**General description**: The saddle-shaped distalometapodial joint I of Therocephalia resembles that of Gorgonopsia, especially in the basal forms such as *Glanosuchus* CGS RS 424 (Fig. 11A and B) and the therocephalian NMQR 3530. However, dorsally, it shows a much lower relief in the more derived forms like *Theriognathus* NHMUK R 5694 (Fig. 12) and *Ictidosuchoides* CGS CM 86–655 (Fig. 11C). Ventrally, the saddle joint is well developed, particularly in the ventral or lateroventral convexity of distale I and in the concavity in the ventral part of the proximal terminus of metapodiale I (Figs. 12 and Table 4). However, the concavity of metapodiale I is not as deep as in Cynodontia. Thus, the morphology of the distalometapodial joint I in *Theriognathus* is intermediate between that of the gorgonopsian saddle joint and the cynodontian pivot joint (see below). Like in Cynodontia the entire joint was more symmetrical in the parasagittal plane.

**Manus of** ***Theriognathus*** **NHMUK R 5694:** The distalometapodial articular facets in the manus are completely exposed (Fig. 12). Distale I shows some fractures or cracks on its ventral face.

At its distodorsal margin, the distal joint facet of distale I bears a transversally orientated lip. This lip is nearly straight and does not show the dorsomedial convexity, which is typical for the distalometapodial joint I in Gorgonopsia. However, the top of this ridge is either broken medially (post-mortem) or was cartilaginous, which is concluded from its porous bone surface. Presumably the dorsomedial side of the lip originally was longer distoproximally, bearing a faint dorsomedial convexity. Laterally, the lip forms a ridge, as is the case in the pes of *Gorgonops* BP/1/4089, but in *Theriognathus* it is relatively smaller when compared with the latter. The nearly transversely orientated concavity of distale I is broader and deeper than in the pes of *Gorgonops* BP/1/4089. The length of the ventral convexity is approximately 13% of the ventral length of distale I (Table 4).

The concavity of metapodiale I trends dorsoventrally. Because the proximolateral convexity is partly damaged, it cannot be determined whether or not the concavity continued into the dorsolateral part of the facet. Such a continuation would be likely because the corresponding convex facet on the distale I is present.

It is not possible to estimate the degree of dorsal extension due to the damaged face on the dorsal border of the articular facet of distale I. However, the presence of the dorsolateral convex ridge on distale I shows that some dorsal extension was possible. From a dorsally extended position, metapodiale I could be flexed in the distalometapodial joint I, but there was very little ventral flexion possible in that joint (about 10°). Medial abduction of about 20° could also take place (Table 4).

Metapodiale I could have been circumducted around the ventral convexity of distale I. The circumduction of metapodiale I in the lateral direction is composed of an abduction of about 25° and a simultaneous rotation of about 20° in the lateral direction. During this movement metapodiale I could be flexed up to approximately 10° ventrally. In the medial direction the circumduction resulted in an abduction of approximately 20°, and a simultaneous ventromedial rotation of approximately 20° was possible. During the medially directed circumduction, flexion of metapodiale I to the zero position was possible (Table 4).

**Manus of** ***Glanosuchus*** **CGS RS424 and pes of** ***Glanosuchus*** **SAM-PK-12051**: The manus is exposed in dorsal and ventral aspects (Figs. 11A and B), but the pes is only exposed in dorsal aspect.

The length of the dorsomedial convexity of distale I in the manus of *Glanosuchus* CGS RS424 is about 17% and in the pes of *Glanosuchus* SAM-PK-12051 it is about 24% of the complete length of the dorsal side of distale I (Table 4). The ventral convexity of distale I in *Glanosuchus* CGS RS424 is flat distally and is raised to a lip with a sharp margin ventrally. The saddle-shaped surface of metapodiale I, however, shows pronounced relief proximally. As can be judged from the distalometapodial joint I in dorsal view, the mobility of metapodiale I was similar to that of Gorgonopsia.

**Manus of** ***Ictidosuchoides*** **CGS-CM86-655**: The right manus is seen in dorsal view (Fig. 11C), and the left from ventral.

Like in *Theriognathus*, the dorsomedial convexity of distale I is tiny. It only reaches about 7% of the dorsal length of distale I (Table 4). Because lateral circumduction is not restricted by a dorsomedially protruding convexity, it is very likely that in *Ictidosuchoides* metapodiale I could be circumducted not only in a medial direction, but also in the lateral direction like in *Theriognathus*.

**Manus of** ***Microgomphodon*** **SAM-PK-K10160**: The manus is exposed completely in ventral and partially in dorsal aspects. The bones are impacted and the possibly cartilaginous joint surfaces are completely missing. Because the distalometapodial joint I of the specimen does not yield any reliable information, it will not be further described here.

**Mobility**: Because the dorsal face of the distalometapodial joint I of *Glanosuchus* is very similar in both manus and pes, the mobility of digit I in manus and pes must have been almost identical, only the amount of the excursion angle might have been slightly different.

In the manus of *Theriognathus*, circumduction in a lateral direction was possible to the same extent as circumduction in a medial direction. The amount of circumduction of metacarpale I in *Ictidosuchoides* cannot be ruled out in this specimen. However, a slight lateral circumduction of metacarpale I in the distalometapodial joint I is preserved *in situ* in *Ictidosuchoides* CGS CM86-655 (Fig. 11C). The mobility of metapodiale I probably was symmetrical in *Ictidosuchoides* because the dorsomedial convexity of distale I is vestigial. Besides circumduction, dorsal extension, ventral flexion and abduction also were possible in the distalometapodial joint I.

#### Non-mammaliamorph Cynodontia

**General description**: In Therocephalia and especially in Cynodontia, the dorsomedial convexity and the dorsolateral ridge of distale I as seen in Gorgonopsia are reduced in earlier forms or completely replaced by a homogenous dorsal margin in later forms (Table 4), which stabilized the metapodiale I from above. This evolution is accompanied by an option for a more laterally directed circumduction of metapodiale I.

**Figure 13:**
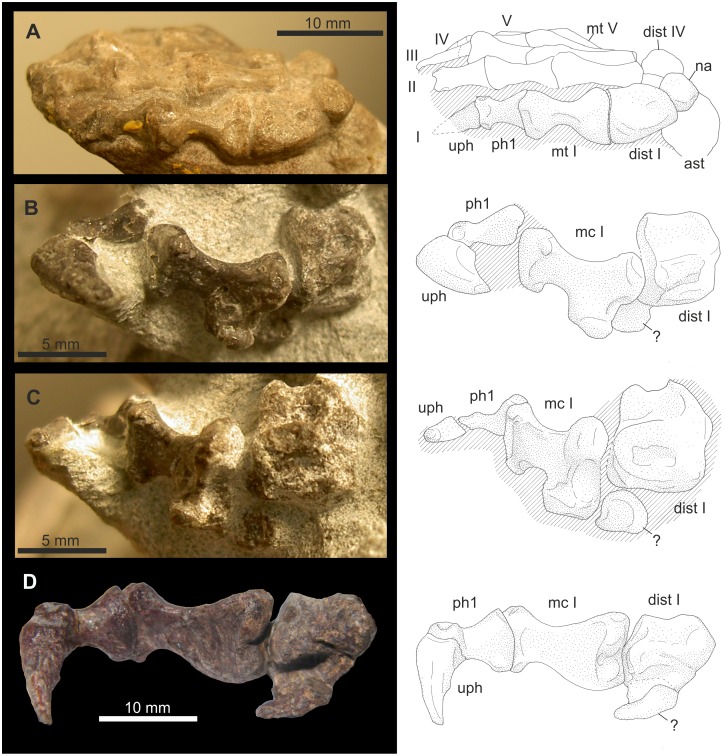
First ray in Cynodontia. A: *Galesaurus* BP/1/4506: Right pes seen mediodorsally. B and C: *Galesaurus* SAM-PK-K10468: Ray I of the left manus in lateroventral (B) and proximoventral views (C). D: *Chiniquodon* PVL 3820: Ray I of the right (?) manus (?) in medioventral aspect (photo courtesy of F. Abdala). Abbreviations: **ast** astragalus, **dist** distale, **mc** metacarpale, **mt** metatarsale, **na** naviculare, **ph** phalanx, **uph** ungual phalanx.

**Figure 14:**
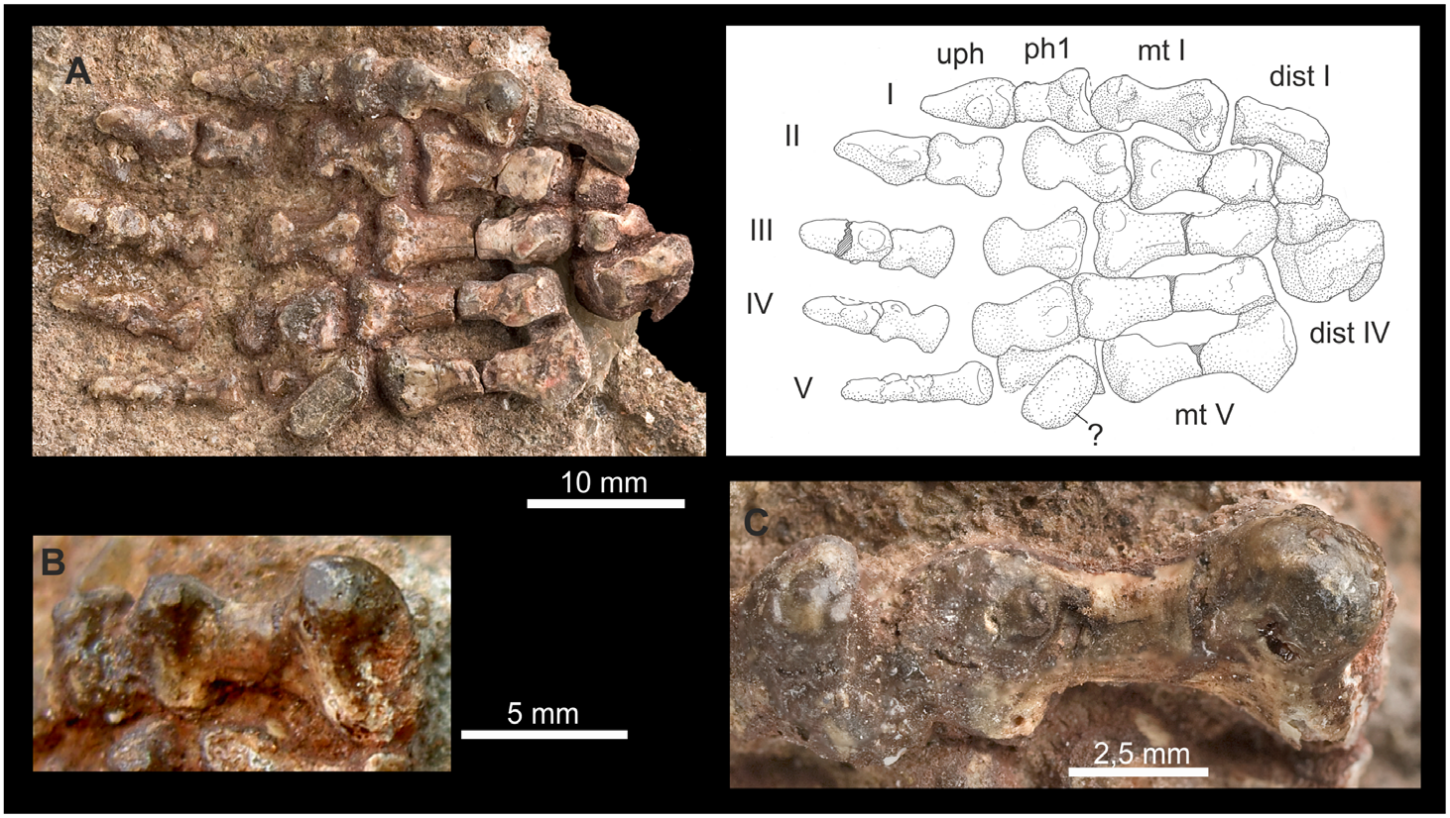
Hallux of *?Scalenodon* NHMUK R 9391. Right pes in ventral aspect (A). First ray of the right pes in proximoventral (B) and in medioventral views (C). Photo A and C courtesy of NHMUK. Abbreviations: **dist** distale, **mt** metatarsale, **ph** phalanx, **uph** ungual phalanx.

In Cynodontia the convexity at the ventral margin of the distal articular facet of distale I served as an articular peg (Figs. 13B–D). This structure was present in Cynodontia but mostly consisted of cartilage (see below). In contrast, the ventroproximal concavity on the proximal facet of metapodiale I is deep, at least in the pes of ?*Scalenodon* NHMUK R 9391 and the manus of *Galesaurus* and *Chiniquodon,* forming a well-defined socket (Figs. 13B–D and 14). Thus, the distalometapodial articulation I in Cynodontia is a peg and socket joint and contrasts with the respective asymmetrical saddle joint of *Titanophoneus* and the near-symmetrical saddle joint of Gorgonopsia and Therocephalia. Evidently, in Cynodontia the dorsal part of the former saddle joint was reduced.

**Metatarsale I of** ***?Scalenodon*** **NHMUK R 9391**: The proximal joint of metatarsale I is completely visible (Fig. 14). The joint surface of the distal part of distale I is eroded.

A large, deep concavity is situated slightly lateral to the centre on the ventroproximal articular face of metatarsale I. There is no dorsomedial depression or dorsolateral concavity. In relation to the proximal width of metapodiale I, the width of the ventromedial convexity is approximately 37% and the ventrolateral convexity is about 20% (Fig. 14B). Dorsally, these convexities are connected by a lip.

The excursion angles of metatarsale I cannot be reconstructed completely, because the articular facet of distale I is badly preserved. However, as can be judged from the articular face of metapodiale I, distalometapodial joint I mainly functioned as a pivot joint, allowing medial and lateral circumduction. The circumduction of metatarsale I in the pes of *?Scalenodon* in medial direction could have been larger than in the manus of *Theriognathus* NHMUK R 5694, because the proximoventral concavity of metapodiale I of *?Scalenodon* is deeper than in the latter.

**Other non-mammaliamorph Cynodontia:** Distale I of *Procynosuchus* TSK 34 is free of matrix, however highly compacted. Metapodiale I is missing. In *Thrinaxodon* BP/1/2776 and *Galesaurus* SAM-PK-K10468 (Figs. 13B and C) the distalometapodial joint I is visible in ventral view with the joint surfaces of distale I partially exposed. On the surface of distale I of *Thrinaxodon* BP/1/2776 the spongy bone structure is visible on almost the entire surface. Apparently some bone or cartilage must have been damaged post-mortem and is now missing. The proximal terminus of metacarpale I is flat in proximal aspect. Likely, the entire articular cap is missing. In *Chiniquodon* PVL 3820 the distalometapodial joint I is completely free of matrix and well ossified (Fig. 13D). However, it was only investigated on photographs. In all the other non-mammaliamorph Cynodontia investigated, the respective joint is visible either in dorsal or ventral aspect.

A shallow or rudimentary dorsomedial convexity on the distodorsal margin of distale I is likely present in Cynodontia, because the dorsomedial part of this margin is usually a little longer than its dorsolateral part (Fig. 13A). But, in the fossil Cynodontia examined here the distodorsal margin of distale I is either partially hidden or compressed. Therefore, it is difficult to say whether or not the dorsomedial part of this articular face shows the same convexity that is seen in Gorgonopsia. If there was such a convexity, it was certainly less pronounced than in Gorgonopsia, because there is no depression visible on the dorsomedial part of the facet of metapodiale I (e.g. manus of *Galesaurus* BP/1/2513a and pes of *?Scalenodon* NHMUK R 9391). In the manus of basal Cynodontia (*Procynosuchus* RC 92, TSK 34 and *Galesaurus* BP/1/2513a) the length of the medial part of the distodorsal margin of distale I is 14–17% longer than its lateral margin, measured in percent of dorsal length of distale I (Table 4). In the manus of *Diademodon* NHMUK R 3581, however, the distodorsal margin of distale I has the same height medially and laterally. In the pedes the dorsomedial part of the distodorsal margin of distale I has a length of approximately 6% of the dorsal length of distale I in *Galesaurus* BP/1/4506 and about 7% in *Diademodon* NHMUK R 3581 (Table 4). Thus, in the more derived forms the dorsomedial elongation of distale I is less pronounced and probably disappeared completely as is seen in the manus of *Diademodon* NHMUK R 3581. In those fossils where it is absent, the distalometapodial joint I is no longer a saddle joint like in Gorgonopsia, but a pivot joint.

In non-mammaliamorph Cynodontia the length of the ventrally or lateroventrally situated convexity of the joint facet of distale I could only be measured in three specimens: in *Galesaurus* SAM-PK-K10468, with an amount of about 5% of the ventral length of distale I; in *Thrinaxodon* BP/1/2776, with a value about 20%; and *Chiniquodon* PVL 3820, with 19% (Table 4). The value of *Thrinaxodon* BP/1/2776 is not accurate due to the bad preservation state of the joint facet. In *Galesaurus* SAM-PK-K10468 (ventral aspect), *Galesaurus* BP/1/2513a (dorsal aspect) and *Chiniquodon* PVL 3820 the pivot joint-character is visible in the proximal facet of metacarpale I. A deep ventroproximal concavity is present, but the concavity does not reach the dorsal part of the facet. The peg-like ventral convexity of distale I in *Galesaurus* SAM-PK-K10468 is very small compared with the ventroproximal concavity of metapodiale I. This is likely due to the partly cartilaginous nature of this convexity in some specimens.

**Figure 15:**
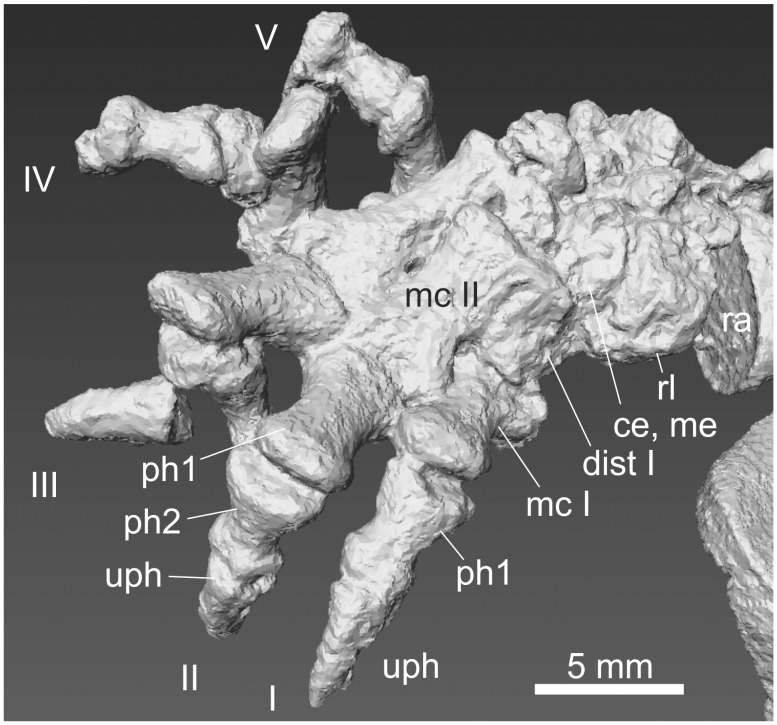
Manus of *Thrinaxodon* BP/1/5558. Right manus in mediodorsal aspect (scan courtesy of F. Abdala). Abbreviations: **ce** centrale, **dist** distale, **mc** metacarpale, **me** medial, **ph** phalanx, **ra** radius, **rl** radiale, **uph** ungual phalanx.

**Mobility**: In the distalometapodial joint I of Cynodontia, the mobility of metapodiale I mainly comprises medial and lateral circumduction. Dorsal extension was possible, and circumduction may have started in a dorsally extended position, and then would have resulted in flexion and abduction during circumduction. Lateral circumduction was constrained by digit II. Therefore, it is likely that the excursion angle was higher in the medial direction than the lateral. Slight lateral circumduction of metapodiale I in the distalometapodial joint I preserved *in situ* is seen in *Thrinaxodon* BP/1/5558 (Fig. 15). Circumduction in a lateral direction was certainly possible to a higher degree than in Gorgonopsia, in which the dorsolateral ridge of distale I is more convex (see above). Circumduction around a dorsomedial convexity of distale I, as is reconstructed for Gorgonopsia, was reduced or even impossible, like in *Diademodon* NHMUK R 3581. Thus both, medial abduction and medial rotation of metapodiale I in maximal dorsal extension was less possible in Cynodontia than in Gorgonopsia.

Ventral flexion might have been possible to some degree, but certainly was restricted, because the facet of distale I is bordered by a sharp frill ventrolaterally and ventromedially (Figs. 13B–D).

In two different specimens of *Galesaurus* of nearly the same size (BP/1/2513a and BP/1/4506), the relief of the distalometapodial joint I in dorsal aspect is higher in the manus than in the pes, but in *Diademodon* NHMUK R 3581 it is the reverse. The mobility of manus and pes in non-mammaliamorph Cynodontia seems to have been very similar. Differences refer to the degree of the excursion angles of metapodiale I in the distalometapodial joint I.

#### Mesozoic Mammaliamorpha

The form of the distalometapodial joint I is highly variable within Mesozoic Mammaliamorpha. As a consequence, the mobility must have been more variable within this group than within non-mammaliamorph Therapsida. In some forms of Mesozoic Mammaliamorpha, the distalometapodial joint I is very wide. In relation to the width of the shaft of metapodiale I the joint of Mesozoic Mammaliamorpha is as wide as that in non-mammaliamorph Cynodontia or even wider. Therefore, the distalometapodial joint I is not rudimentary but strong and functional. However, there is a change in function, since circumduction is not necessarily part of the outfit of the joint as it is in Therapsida (*sine* Dicynodontia).

**Figure 16:**
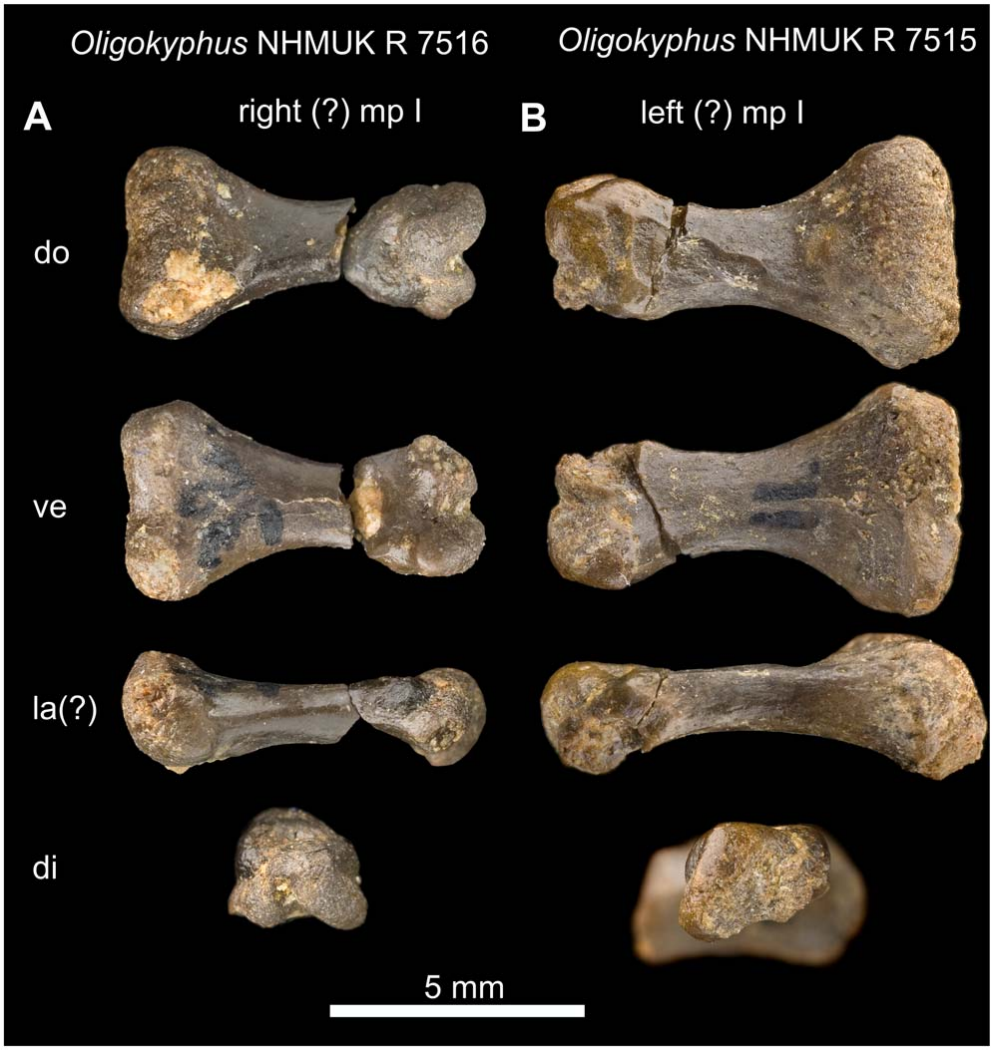
First metapodiale of *Oligokyphus*. A: *Oligokyphus* NHMUK R 7516: Metapodiale I, probably of the right autopodium. B: *Oligokyphus* NHMUK R 7515: Metapodiale I, probably of the left autopodium. Photos courtesy of NHMUK. Abbreviations: **di** distal, **do** dorsal, **la** lateral, **mp** metapodiale, **ve** ventral.

***Oligokyphus*** **NHMUK R 7515, 7516 and** ***Eozostrodon*** **CAMZM Eo PC:** Both metapodialia I are free of matrix (Fig. 16).

The proximal articular end of metapodiale I (manus or pes?) of *Oligokyphus* NHMUK R 7515 measures 2.56 times the width of the shaft, and in metapodiale I (manus or pes?) of *Eozostrodon* it is even wider (Table 5). This articular facet is wide compared with that of non-mammaliamorph Cynodontia, which show values in the manus and pes between 1.20 and 2.41 times the width of the shaft. This is insofar noteworthy, because the entire autopodium becomes more slender and gracile in Mesozoic Mammaliamorpha when compared with the cynodont predecessors, but by contrast the distalometapodial joint I becomes wider. Due to its wide proximal articular end, metapodiale I cannot be aligned with metapodiale II, but is still slightly separated even in the most adducted position. In *Eozostrodon* and *Oligokyphus*, the proximal articular facet of metapodiale I is dorsoventrally convex and transversally nearly straight. Only in *Oligokyphus* is a shallow concavity present on its ventroproximal side (Fig. 16). Flexion and extension were possible in this joint in both genera. Distale I remains unknown in both specimens. If the morphology of the articular faces was not completely different from the bony face due to a cartilaginous covering, there was no or only a very small amount of circumduction and abduction of metapodiale I possible in this joint.

**Table 5:** Ratio between the proximal width of metapodiale I and the width of the middle part of the shaft.

taxon	m	p	m or p
*Oligokyphus* NHMUK R 7515			2.56
*Eozostrodon* CAMZM Eo PC			2.75
*Eozostrodon* CAMZM Eo PC			3.25
*Eozostrodon* CAMZM Eo PC			3.50
*Erythrotherium* SAM-PK-K359		1.00	
*Megazostrodon* NHMUK M 26407		2.20	
*Jeholodens* GMV 2139a	2.00	1.13	
*Gobiconodon* MCZ 19860			2.00
*?Eucosmodon* AMNH 16325		1.59	
*Zhangheotherium* IVPP V7466	1.41		
*Zhangheotherium* CAGS-IG-97.07352		2.80	
*Henkelotherium* Gui Mam 138/76	1.96		
*Sinodelphys* CAGS00-IG03		3.33	
*Asiatherium* PIRAS 3907	2.00		
*Eomaia* CAGS01-IG-1a	2.33	1.78	

Abbreviations: **m** manus, **p** pes.

**Figure 17:**
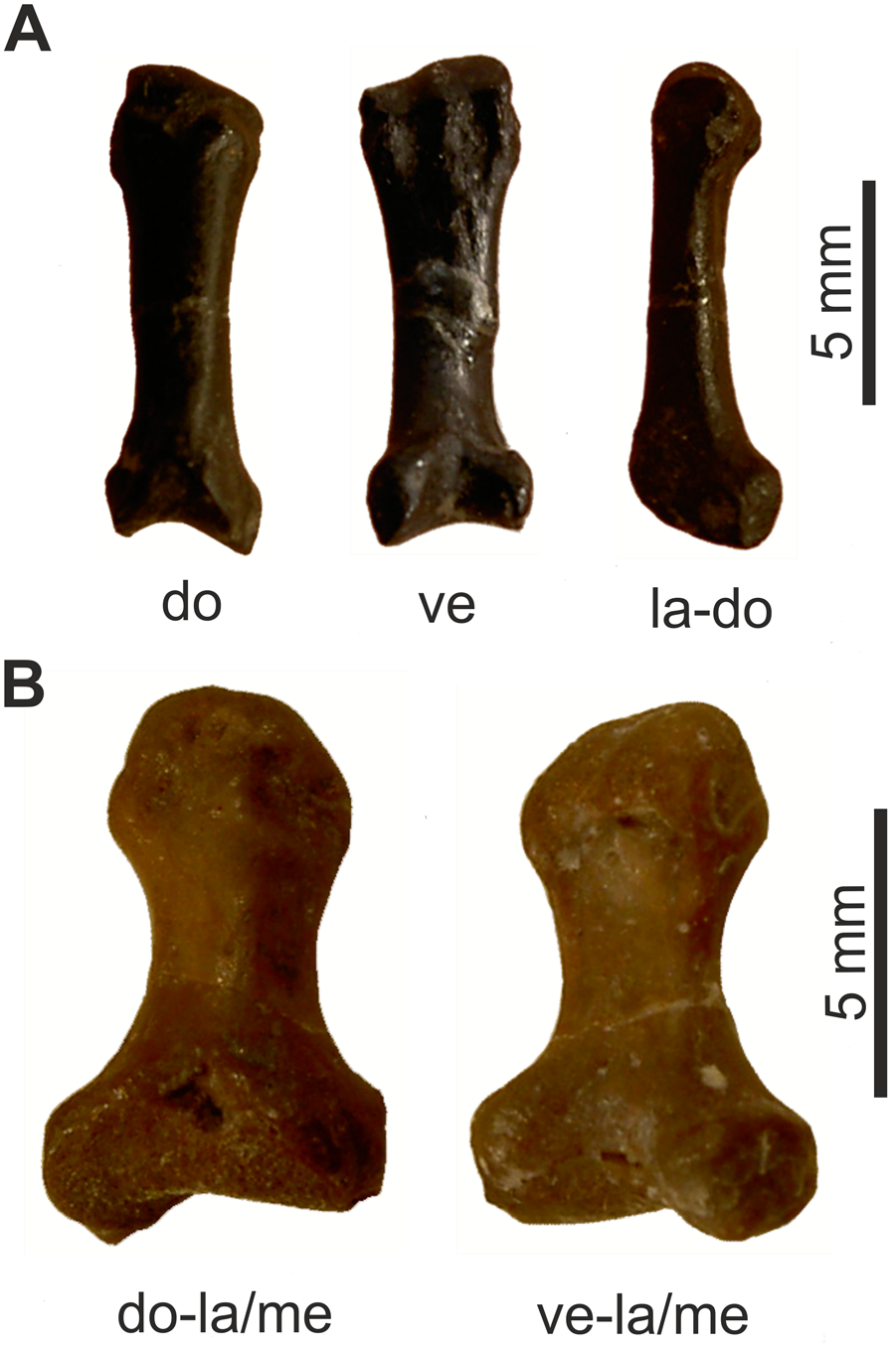
Two mammalian metapodialia I. A: *?Eucosmodon* AMNH 16325: First metapodiale, which was assigned to the right pes by Granger and Simpson [14]. B: *Gobiconodon* MCZ 19860: First metapodiale. Abbreviations: **do** dorsal, **la** lateral, **me** medial, **ve** ventral.

***Gobiconodon*** **MCZ 19860,** ***?Eucosmodon*** **AMNH 16325 and** ***Kryptobaatar*** **ZPAL MgM-I/41**: In *Gobiconodon* and *?Eucosmodon* the metapodialia are freed of matrix (Fig. 17). The distalometapodial joint I of *Kryptobaatar* ZPAL MgM-I/41 has been described by Kielan-Jaworowska and Gambaryan ([13], p. 80–81). In the fossil, it is visible in dorsal and medial aspect.

In *Gobiconodon* the width of the proximal articular end of metapodiale I is twice the diameter of the shaft. In *?Eucosmodon*, it is only 1.6 times as wide as the shaft (Table 5). The proximal articular end of metapodiale I (manus or pes?) of *Gobiconodon* and in the pes of *?Eucosmodon* is a saddle joint, which shows a concavity extending dorsoventrally (Fig. 17). In contrast to the case in non-mammaliamorph Cynodontia, the concavity extends onto the dorsal part of the joint, where it is flatter as it is on the ventral side, but still deep. Prominent laterally and medially situated convexities occur on the proximal articular end of metapodiale I in *Gobiconodon*. In *?Eucosmodon*, these convexities are subspherical in outline. They are rounded towards the articular facet and flattened on the lateral and medial sides of the bone; here there are also insertion pits for lateral tendons. Therefore, in contrast to the joint of *Gobiconodon*, that of *?Eucosmodon* is intermediate between a saddle and a hinge joint.

Both flexion and extension, as well as ab- and adduction, were possible in the distalometapodial joint I of *Gobiconodon* and *?Eucosmodon*. Very likely some circumduction could also have taken place, especially in *Gobiconodon*. For *?Eucosmodon*, Granger and Simpson [14] suggest that a small amount of opposability of metapodiale I was possible.

The distalometatarsal joint I of *Kryptobaatar* resembles that of *?Eucosmodon*, but the convexities of the proximal facet of metatarsale I are more rounded. According to Kielan-Jaworowska and Gambaryan ([13], p. 80–81) the excursion angle of metatarsale I of *Kryptobaatar* was smaller during abduction than in *?Eucosmodon*. They describe this joint in *Kryptobaatar* as a hinge joint. However, according to the terminology applied here, it is a saddle joint.

**Figure 18:**
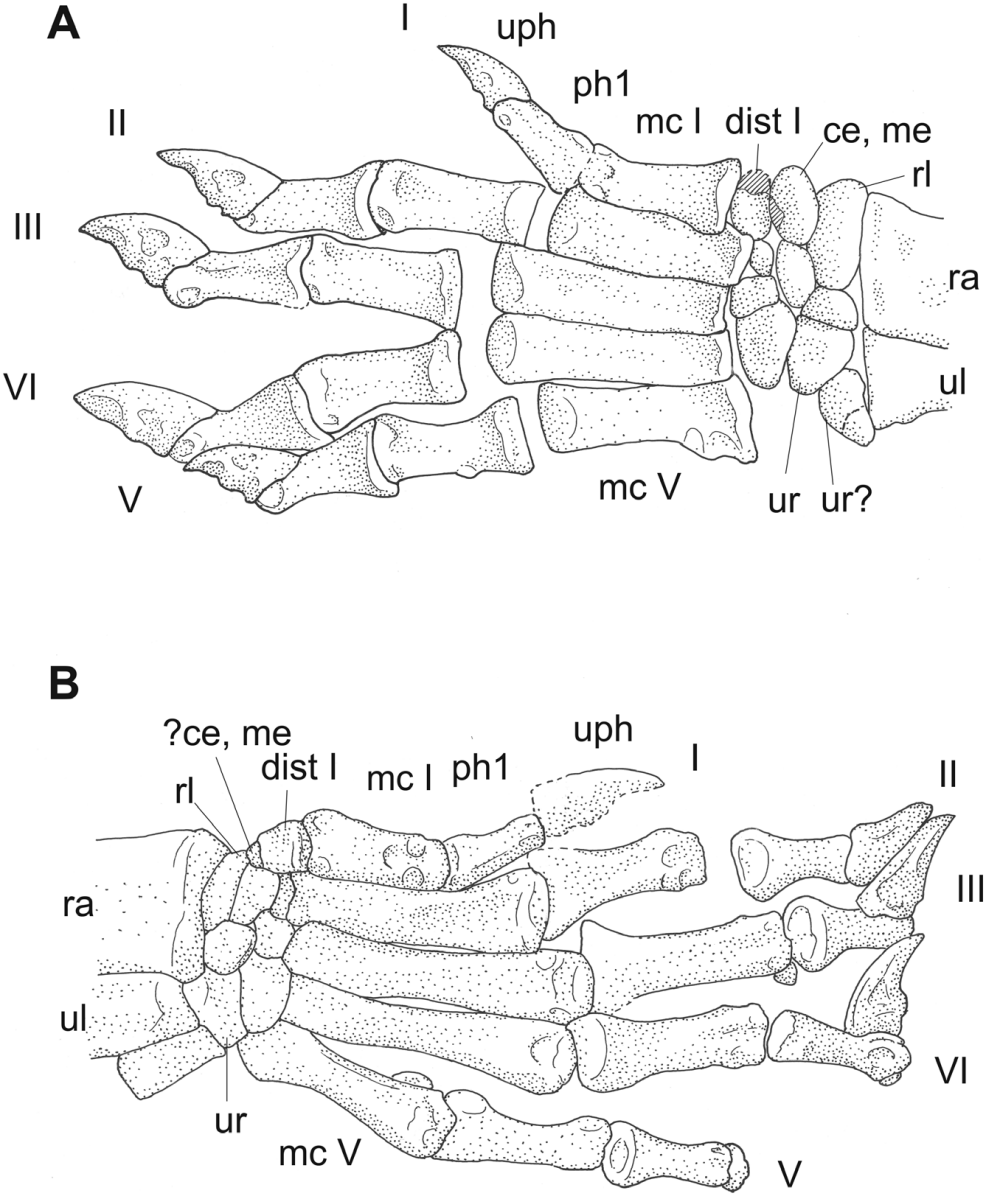
Manus of two Jehol Mammalia. A: *Jeholodens* GMV 2139a: Left manus, mainly in dorsal view; middle and ungual phalanges in dorsolateral aspect. B: *Zhangheotherium* IVPP V7466: Right manus mainly from dorsal, unguals in lateral or medial view. Abbreviations: **ce** centrale, **dist** distale, **mc** metacarpale, **me** medial, **ph** phalanx, **rl** radiale, **ra** radius, **ul** ulna, **uph** ungual phalanx, **ur** ulnare.

**Figure 19:**
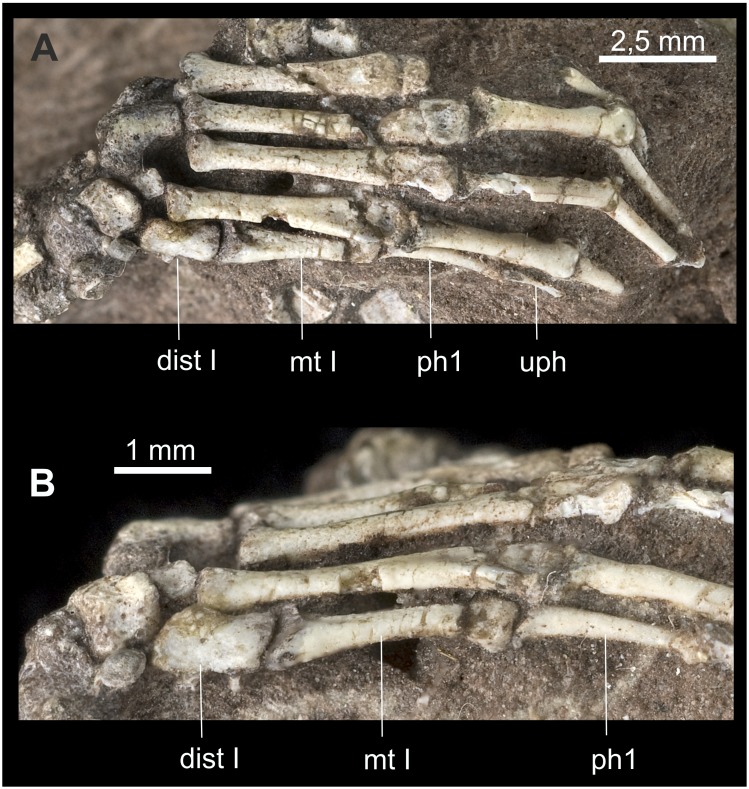
Hallux of *Megazostrodon* NHMUK M 26407. Left pes in dorsal (A) and dorsomedial (B) aspect (photos courtesy of NHMUK). Abbreviations: **dist** distale, **mt** metatarsale, **ph** phalanx, **uph** ungual phalanx.

**Other Mesozoic Mammaliamorpha**: Distalometapodial joint I is visible in dorsal view in the manus and pes of *Jeholodens* GMV 2139a (Fig. 18A) and *Eomaia* CAGS01-IG-1a (cast), in the manus of *Henkelotherium* Gui Mam 138/76, *Zhangheotherium* IVPP V7466 (Fig. 18B) and *Asiatherium* PIRAS 3907 (cast) as well as in the pes of *Megazostrodon* NHMUK M 26407 (Fig. 19) and *Zhangheotherium* CAGS-IG-97.07352 (cast). The distalometapodial joint in the pes of *Zhangheotherium* IVPP V7466 is seen in ventral aspect.

The basal width of metapodiale I in relation to the width of its shaft is smaller in these specimens than in *Eozostrodon* and *Oligokyphus* (Table 5). In those specimens, which are preserved in articulation, distale I and metapodiale I nearly parallels metapodiale II. However, Hu et al. [16] concluded from the position of the carpals that *Zhangheotherium* had a ‘spreizhand’, a manus with diverging metapodialia I and II.

In dorsal aspect the distalometapodial joint I is slightly convex distally (manus and pes of *Zhangheotherium* (Fig. 18B), pes of *Megazostrodon* (Fig. 19) and manus of *Asiatherium*) or slightly concave (pes of *Eomaia*). For *Henkelotherium*, Krebs [15] concluded (translated from German): “Metacarpale I (…) differs from the other metacarpalia by its stronger proximal widening. This articular terminus possesses a medial and a lateral condylus with a depression lying in between them.” ([15], p. 75).

The excursion angles of the abduction and circumduction movements – as far as these are reconstructed in dorsal view – might have been smaller in these species than in *Gobiconodon* and *?Eucosmodon*, with their pronounced saddle joint. Hu et al. [16] confirm a restricted mobility in the pollex of *Zhangheotherium* IVPP V7466: “The pollex and the digit V are somewhat separate from other digits, but there is no evidence supporting opposable pollex and grasping ability.” ([16], p. 121). However, the width of the joint with respect to the shaft of metapodiale I shows that the joint is very stout and at least was functional in flexion, extension and some abduction.

### 2 Metapodiophalangeal Joint I of Manus and Pes

**General description**: In non-mammaliamorph Therapsida (*sine* some Anomodontia) the metapodiophalangeal joint I resembles that of the middle joints of digits II–V in dorsal aspect (Fig. 15). Distodorsally, the terminus of the head of metapodiale I is either marked by a distally curved sharp lip or a distally pointing protrusion (e.g. Figs. 6 and 11C). The articular facet faces distoventrally, like that of the basal phalanges of the lateral digits. However, the morphology of the articular facet differs from that of the basal phalanges of digits II–V, resulting in functional differences (for details see below). Due to the protrusion or lip and the distoventral orientation of the joint facet, dorsal extension of metapodiale I was blocked in the metapodiophalangeal joint I of non-mammaliamorph Therapsida (*sine* some Anomodontia). The consequence is that ray I was held flexed in this joint forming a digital arcade. This is also the case in the lateral digits of most Therapsida [22].

In Mesozoic Mammaliamorpha, however, a digital arcade in ray I was absent, which is concluded from the option for dorsal extension of the first phalanx in the metapodiophalangeal joint I in this group (see below).

#### Dinocephalian *Titanophoneus*

In the manus and pes of *Titanophoneus* PIN 157/1 the head of metapodiale I possesses a distally pointing protrusion on its distodorsal margin, like in the basal phalanges of the lateral digits (Figs. 6 and 7). As observed in the basal phalanges of digit II–V, the joint facet of the head of metapodiale I faces distoventrally at an angle of 55° to the long axis of the shaft. However, unlike the middle joints of digits II–V, this joint is a saddle joint [1]: In ventral view the distodorsal protrusion of metapodiale I forms one convexity of the saddle. The second emerges from the proximoventral margin of the joint. Both convexities are connected by a vertical ridge, which is slightly concave in its long axis. The medial flank of the ridge is flatter than the lateral. The proximal articular facet of the first phalanx I possesses a dorsoventrally extending concavity (Fig. 7A).

In dorsal aspect, the metapodiophalangeal joint I is nearly identical in manus and pes. In ventral view, however, its relief in the manus is stronger than in the pes (Figs. 7A and B). Phalanx I is twisted along its long axis. Compared with the proximal joint, the distal one is rotated in lateral direction at about 25° in the manus and about 8° in the pes.

Phalanx 1 has three degrees of freedom in that joint: flexion and extension, ab- and adduction and partial rotation in lateral and medial direction. From the zero position, ventral flexion was possible to about 70°. Dorsal extension was blocked by the distodorsal protrusion of metapodiale I. In the extended joint, the first phalanx could be abducted about 10° in a lateral direction and about 16° in a medial direction. During flexion, the capacity of medial abduction increased to about 20°. In the flexed position, the coherence of the joint facets in medial abduction is very high, but it is reduced in lateral abduction. Flexion and medial abduction of the first phalanx were inevitably combined with a lateral rotation of 20°, because the joint facet of metapodiale I is longer proximodistally in its central part than medially.

In the standard position phalanx 1 was flexed in the metapodiophalangeal joint I to about 25°.

#### Eupelycosaurian *Dimetrodon* CAMZM T 857 (manus or pes) and MNG 10654 (pes)

The head of metapodiale I is similar to that of *Titanophoneus* (Figs. 5A–C). The articular facet is strongly asymmetric, with a steep lateral flank and a flat medial one. Metatarsale I and first phalanx I in the pes of *Dimetrodon teutonis* MNG 10654 are preserved *in situ*, with ray I flexed to a digital arcade in the metatarsophalangeal joint I (Fig. 5A). This position approximates the standard position with a flexion of phalanx 1 of about 40°.

#### Gorgonopsia

**Figure 20:**
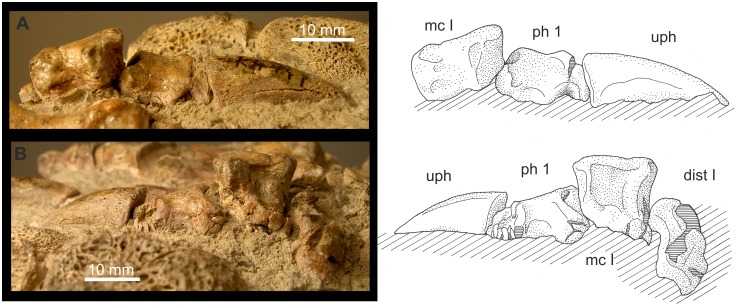
Gorgonopsian SAM-PK-K4441: First ray of the right manus. Lateral (A) and medial aspect (B). Abbreviations: **dist** distale, **mc** metacarpale, **ph** phalanx, **uph** ungual phalanx.

In the manus of Gorgonopsia indet. SAM-PK-K4441, the slightly damaged metapodiophalangeal joint I appears to be saddle-shaped, like that in *Titanophoneus* (Fig. 20). The angle between the articular facet and the shaft is a little higher than in *Titanophoneus*, which means that in the standard position phalanx 1 was less flexed (20°) in the metapodiophalangeal joint I.

In *Gorgonops* BP/1/4089 and Gorgonopsia indet. BP/1/600 the joint is preserved in articulation, and thus the facet is only visible in dorsal and ventral view, strongly suggesting the saddle shape of the joint (Figs. 8, 9D and E). In the standard position a flexion of phalanx 1 in the metapodiophalangeal joint I of about 30° is very likely.

#### Therocephalia

In *Theriognathus* NHMUK R 5694, the bony surface of the distal articular facet of metacarpale I is mostly missing. As can be judged from the preserved form, the articular facet morphology is as follows: the head of metacarpale I shows a distally pointing distodorsal protrusion (Fig. 12), which is lacking in the basal phalanges II–V of the same specimen. However, a similar protrusion occurs in the basal phalanges of the manus and pes of *Titanophoneus*. Also ventroproximally the articular facet of metapodiale I of *Theriognathus* resembles the basal phalanges II–V of *Titanophoneus* [22]. Ventroproximal to the protrusion, the joint surface is distoproximally concave, forming a short saddle joint. Further ventroproximally, the joint surface divides into two equally sized slightly convex articular facets, situated laterally and medially and separated by a depression. In contrast to the basal phalanges II–V, the entire articular face on the distal end of metacarpale I is relatively steep, being ventroproximally inclined by about 70° (Fig. 12).

Flexion of phalanx 1 was possible, but because of the preservation state, the angle cannot be reconstructed. In the standard position ventral flexion of phalanx 1 seems to be only 10°, but this value is tentative, because of the bad preservation state of the joint. When extended, phalanx 1 could be abducted in the lateral direction to about 20°. This movement was inevitably combined with a circumduction about 10° in the medial direction. Medial abduction was only possible up to about 13°, but the joint contact was more stable than during lateral abduction. Lateral circumduction inevitably accompanied medial abduction. Abduction was blocked during maximal flexion, where the joint worked like a roller or hinge joint and the coherence of the joint facets was high.

Ray I of *Glanosuchus* CGS RS424 is preserved in articulation so that the joint facets of the metapodiophalangeal joint I are invisible. However, the flexion of phalanx 1 in that joint in the standard position could be estimated to be about 35°.

As far as can be seen in ventral aspect, *Microgomphodon* SAM-PK-K10160 shows a joint structure similar to that of *Theriognathus*. In *Microgomphodon* the joints were partly cartilaginous, as is concluded from the missing articular surface. The joints were compacted during diagenesis.

#### Non-mammaliamorph Cynodontia

The metapodiophalangeal joint I in the manus of *Thrinaxodon* BP/1/2776, BP/1/1737, BP/1/5558 and *Galesaurus* SAM-PK-K10468 and in the pes of *?Scalenodon* NHMUK R 9391 is visible in ventral view (Figs. 13B, C and 14). The joint of *Galesaurus* SAM-PK-K10468 is slightly compacted, and the articular surface is missing. In dorsal view it is seen in *Procynosuchus* RC 92 (manus), *Galesaurus* BP/1/2513 (manus), *Galesaurus* BP/1/4506 (pes; Fig. 13A), *Thrinaxodon* NMQR 628 (probably a pes), *Thrinaxodon* BP/1/1730 (manus), BP/1/5558 (manus; Fig. 15) and *Diademodon* NHMUK R 3581 (manus and pes).

The head of metapodiale I possesses a very weak distodorsal protrusion in *Procynosuchus* and *Galesaurus* (Fig. 13A). In *Thrinaxodon* and *Diademodon*, this protrusion is replaced by a distally convex lip (Fig. 15). The articular facet faces distoventrally. It is ellipsoidal in outline with its long axis orientated mediolaterally (Figs. 13B and 14C). On the proximoventral margin of the ellipsoid joint, a medial depression divides two very short, medial and lateral, convex extensions of the facet. This is similar to the condition in *Theriognathus*, except that the two extensions are shorter. Thus, the joint resembles that of the basal phalanges of the lateral digits in the same species [22], but the distoventral ellipsoidal part is larger and the divided proximoventral part is shorter, especially in *Thrinaxodon*. The proximal facet of the first phalanx I is not preserved in *Thrinaxodon* and not visible in *Galesaurus*. In *?Scalenodon* it is concave in proximal view.

Due to the ellipsoidal distal joint facet of metapodiale I the abduction capacity is higher than in the middle joints of the adjacent digits. A rotation of the first phalanx was also possible, because of the loose joint contact. When fully flexed, however, abduction and rotation was blocked because of the hinge like joint character in the flexed position. The excursion angle of ventral flexion in the pes of *?Scalenodon* is estimated to have been about 90°. Dorsal extension was blocked by the distodorsal lip of metatarsale I. The excursion angle of abduction was less than that of the extension and flexion movement.

In the standard position, phalanx 1 was flexed in the metapodiophalangeal joint I by about 45° in the manus of *Galesaurus* SAM-PK-K10468 and about 40° in the manus of *Thrinaxodon* BP/1/2776 and BP/1/1737, provided the proximal articular face of phalanx 1 was perpendicular to the shaft axis of the bone, which is usually the case.

#### Mesozoic Mammaliamorpha

The distodorsal part of the head of metapodiale I is a joint intermittent between a ball and an ellipsoid joint. It is slightly asymmetric in the pes of *Erythrotherium* and *Megazostrodon*, manus or pes of *Gobiconodon* (Fig. 17B), manus and pes of *Jeholodens*, or highly asymmetric in the manus (?) of *Oligokyphus* NHMUK R 7515, 7516 (Fig. 16), manus or pes of *Eozostrodon* and the pes of *?Eucosmodon* (Fig. 17A). Metapodiale I is exposed in ventral aspect in the manus (?) of *Oligokyphus* NHMUK R 7515, 7516 (Fig. 16), manus or pes of *Gobiconodon* (Fig. 17B) and *Eozostrodon*, and in the pes of *Zhangheotherium* IVPP V7466 and *?Eucosmodon* AMNH 16325 (Fig. 17A). In the manus or pes of *Gobiconodon*, the facet of the distal end of metapodiale I continues distoventrally into a pair of prominent tubercles sitting laterally and medially on the proximal margin of the facet. These tubercles resemble the distoventral tubercles of metapodialia II–V of non-mammaliamorph Synapsida and also some Mammaliamorpha like *Gobiconodon* itself. In the manus (?) of *Oligokyphus* and one metapodiale I of *Eozostrodon*, the joint facet consists of two large oblique condyli, the medial of which is one and a half times as big as the lateral one. However, in *Eozostrodon*, the respective condyli are slightly less pronounced and asymmetric than in *Oligokyphus*. The condyli converge distodorsally, forming the asymmetric ball joint (details for *Oligokyphus* see below). Distoventrally, metapodiale I of *Zhangheotherium* IVPP V7466 bears symmetric, laterally and medially situated condyli, with very low relief. Proximoventral to the condyli is one or probably are two sesamoids that match depressions at the base of the condyli. In *?Eucosmodon* and one metapodiale I of *Eozostrodon*, it is very likely that sesamoids were present during life. This is suggested by depressions ventroproximal to the distoventral condyli, where sesamoids could have articulated (Fig. 17A).

In contrast to the state inferred for most of the non-mammaliamorph Therapsida, dorsal extension of the first phalanx I in the metapodiophalangeal joint I was possible in the Mammaliamorpha considered here. A slight dorsal extension was the standard position of the first phalanx, as is also the case in the basal joints of digits II–V. In its dorsally extended position, the first phalanx had three degrees of freedom: flexion, abduction and rotation. In the articulated skeleton of *Jeholodens*, a slight medial abduction of the first phalanx I is seen (Fig. 18A). During ventral flexion, abduction and rotation were either blocked or constrained by the asymmetry of the joint. This asymmetry resulted in abduction and rotation during the flexion movement, as is further explained in the following section on *Oligokyphus*.

***Oligokyphus*** **NHMUK R 7515, 7516**: Both metapodialia I are free of matrix (Fig. 16). Specimen number 7515 probably represents a left metapodiale, 7516 a right one. This is judged from the base of the bones, where one side is flattened, which likely formed the contact area with metapodiale II, whereas the other side is more convex, which seems to be the medially situated insertion area of strong tendons. Assuming that this assignment to the left or right side respectively is correct, the asymmetry of the distal joint facet of metapodiale I is the same as in *?Eucosmodon* AMNH 16325 (Fig. 17A), where metatarsale I was allocated to the right pes by Granger and Simpson [14]. Because of these reasons, we are taking this assignment to the left or right side as the basis for the following description.

The lateral condyle of the distal joint in NHMUK R 7515 is damaged.

As was described above, the joint is an irregular ball joint in dorsal aspect, the main part of which faces dorsolaterally. Distally and ventrally the joint converts into a hinge joint with two condyli, which are obliquely orientated, diverging ventrally and converging dorsally. The medial condyle is one and a half the size of the lateral condyle and continues without interruption onto the ball joint dorsally. However, the lateral condyle shows a faint constriction at the distal margin of the ball joint. Ventrally the condyli terminate abruptly in a sharp edge, proximally followed by a concave descent to the shaft of the bone. The first phalanx I is unknown.

Flexion and extension were the main actions of the first phalanx in that joint, as far as can be judged from the joint facet of metapodiale I. Dorsal extension was also possible. Ventral flexion was only possible to a maximum of 70°, because the sharp edge of the condyli ventrally would not have allowed a greater excursion angle. Because the medial condyle is bigger than the lateral, the first phalanx must have been laterally abducted about 15–25° in all positions, in which it contacted both condyli, assuming that the proximal facet of the first phalanx was perpendicular to its shaft. Abduction increased during dorsal extension, combined with an inevitable lateral rotation of the first phalanx. In case the first phalanx lost contact with the lateral condyle during maximal extension, then three degrees of freedom would have been possible in that position: flexion, abduction and rotation.

### 3 Terminal Joint in Manus and Pes

The terminal joint of the first phalanx I is a hinge joint and equals in its form and function the terminal joints of digits II–V. The head of the first phalanx I forms a trochlea with a dorsoventrally orientated intercondylar sulcus. The proximal end of the ungual phalanx bears a socket with a dorsoventrally orientated intercotylar ridge.

Flexion and extension were possible in this joint. The excursion angle of the terminal joint probably reached the same range as the terminal joints of digits II–V in the same specimen, ranging between 100° and 170° [17], [22]. In those forms in which the trochlea narrows distodorsally, rotation and abduction could have taken place to a small degree in the dorsally extended position of the ungual phalanx.

## Ichnology

Fossil tracks usually preserve manual and pedal sole morphology and yield some hints to the dynamics of load transfer during the stride. Thus, they can add reasonable information for the reconstruction of the posture of manus and pes during gait and stance. However, in order to investigate the morphology of the soles in detail from the literature, the track must be very well preserved and well documented by shallow light or 3D photography, or by figures, which allow an insight into the relief of the footprint and not just the outline. There are only a few synapsid tracks available that allow the study of the acropodial posture. The best trace fossils of the different evolutionary stages known to us are presented here.

**Figure 21:**
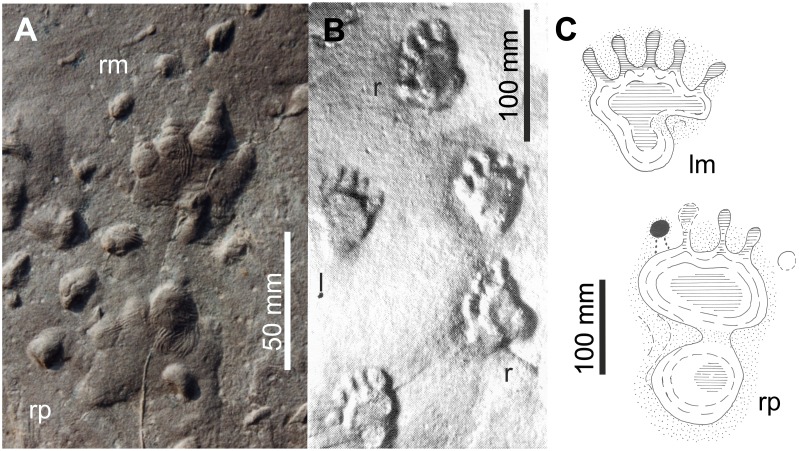
Ichnotaxa of probably non-mammaliamorph synapsid origin . A: *Dimetropus leisnerianus* MNG-13490 from Bromacker, Germany, Early Permian (photograph courtesy of S. Voigt). B: *Gallegosichnus garridoi*, referred to a track of a non-mammaliamorph Therapsida, Los Menucos, Argentina, Late Triassic (photograph courtesy of G. Leonardi, [34] pl. XVI: Fig. 2). C: *Planipes brachydactylus*: Track of a non-mammaliamorph Therapsida, Lodeve, France, Late Permian (redrawn after Gand et al. [35] part of Fig. 17). Abbreviations: **l** left, **m** manus, **p** pes, **r** right.

The track maker of *Dimetropus leisnerianus* MNG-13490 (Fig. 21A) from Bromacker near Gotha (Germany) is referred to the Sphenacodontidae or Caseidae [33]. Impressions of basal pads are aligned in the ray axes of rays II–V [22]. In the case of the first ray, the sole imprint shows a concavity at the base of ray I. A separate terminal pad impression occurs in the first ray in both the manual and pedal tracks. Claw imprints are absent in all rays, but present in other trackways of *Dimetropus leisnerianus* [33]. The distance between the terminal pad imprint in ray I of manus and pes and their sole imprint approximately equals the distance between the terminal and basal pad imprints of ray II. This arrangement indicates that there was not only a digital arcade in digits II–V [22], but also in ray I.

The therapsid tracks *Gallegosichnus garridoi* (Fig. 21B) [34] and *Planipes brachydactylus* (Fig. 21C) [35] also show digital arcades in ray I, with an approximately equal distance between the terminal pad impression and the general sole impression as in the digits II–V.

**Figure 22:**
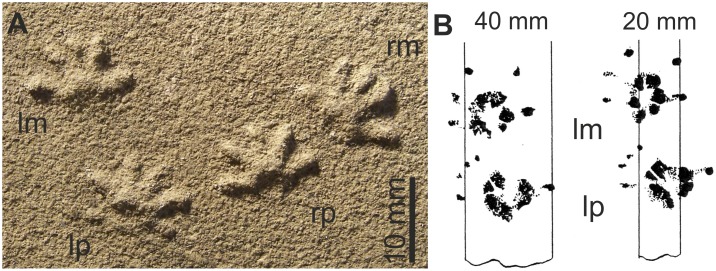
Mammalian Ichnotaxa. A: *Ameghinichnus patagonicus* from Estancia Laguna Manantiaes, Argentina, Middle Jurassic (photograph courtesy of S. de Valais). B: Track of *Didelphis marsupialis* (Figure courtesy of Elsevier, [37] part of Fig. 3, p. 55). Abbreviations: **l** left, **m** manus, **p** pes, **r** right.

In *Ameghinichnus*, the imprints show that the arcades of digits II–V were low, but present (Fig. 22A) [36]. In ray I there is no digital arcade or it was extremely low. Ray I is slightly shorter than ray II–V. From the distal end of the impression to the proximal end of the proximal pad impression the manual ray I is about 67% and 77% of the length of ray II, and in the pedal imprint about 71% and 90%. It is very likely that in ray I the proximal pad was not underlying the distalometapodial joint, but the metapodiophalangeal joint as in *Didelphis*.

In the pes of *Didelphis* the ungual phalanx of digit I bears no claw. The digit terminates in an apical bulb. All the other digits possess claws [37]. A pad below the metapodiophalangeal joint I in manus and pes contacts the substrate during the propulsion phase. The tracks of *Didelphis* show separate imprints of the proximal and the terminal pad of the manual ray I or the proximal pad and the apical bulb in pedal ray I, respectively. Both pad imprints are connected by a faint line not in the left, but in the right trackway, where the diameter of the cylindrical substrate is smaller (Fig. 22B). Manual ray I lacks a digital arcade, which is present in the terminal joint of pedal ray I but not in the metapodiophalangeal joint (photo in [38] vol. I, p. 239). The distance between the distal margins of the pad imprints in ray I in the manus is about 62% and 74% of the pad distance in ray II, in the pes about 75% and 83%. Thus, the loss of the digital arcade in the metapodiophalangeal joint I is inferred from the reduction of the pad distance in the first ray. Thus, it is more than likely that ray I in the track maker of *Ameghinichnus* had undergone such a development.

## Discussion

### 1 Standard Position of Ray I

In the metapodiophalangeal joint I of non-mammaliamorph Synapsida, dorsal extension is anatomically blocked. Some Anomodontia are exceptions in which dorsal extension is possible [17]. When dorsal extension is blocked in the metapodiophalangeal joint I, ray I shows a digital arcade in this joint like in the middle joints of digits II–V [22]. The digital arcade of ray I often is preserved *in situ*; e.g., in the pes of *Dimetrodon* MNG 10654 (Fig. 5A), *Ictidosuchoides* SAM-PK-K10704 (Fig. 11D) and in the manus of *Glanosuchus* CGS RS 424 (Fig. 11A), *Ictidosuchoides* CGS CM 86–655 (Fig. 11C), *Zorillodontops* and *Thrinaxodon* BP/1/5558 (Fig. 15). Watson [39] described a probable digital arcade in ray I of *Ericiolacerta parva* CAMZM T 369: “The short, slender first metatarsale is turned up dorsally to the remainder of the foot exactly as is the first metacarpale. The occurrence of this curious displacement in both fore and hind feet can scarcely be entirely accidental; it must imply that the thumb and big toe possessed some independence and were moveable as units….” ([39], p. 1178). In the standard position the degree of flexion of phalanx 1 in the metapodiophalangeal joint I varies between 10° and 45° (manus of *Titanophoneus* PIN 157/1: ∼25°, gorgonopsian SAM-PK-K4441: ∼20°, *Glanosuchus* CGS RS424: ∼35°, *Theriognathus* NHMUK R 5694: ∼10° (?), *Galesaurus* SAM-PK-K10468: ∼45°, *Thrinaxodon* BP/1/2776 and BP/1/1737: ∼40° and pes of *Dimetrodon* MNG 10654: ∼40° and *Gorgonops* BP/1/4089: ∼30°).

In Synapsida with a digital arcade in ray I, the main load transferring elements were either the proximal part of metapodiale I together with the underlying pad and the terminal pad, or the load was directly transferred to the terminal pad, underlying the terminal joint and the flexor tubercle of the ungual phalanx. This contrasts with the force transmission system of rays II–V, where, besides the terminal joint and pad, the distal part of the metapodiale and the proximal part of the basal phalanx together with the underlying basal pad took part in the load transfer to the substrate.

In the track record of non-mammaliamorph Synapsida, the digital arcade of ray I is evident, because the middle part of ray I shows no imprint or only a very faint one (Fig. 21). Additionally, the distance between the proximal and the terminal pad in ray I and ray II is equal. The track record shows that the distalometapodial joint I either was underlain by a basal pad, which was somewhat smaller than the basal pads of the lateral digits, or the distalometapodial joint was integrated in the entire sole cushion.

In forms with a digital arcade in metapodiophalangeal joint I, the first phalanx I is similar in its topography, orientation and function to the penultimate phalanges of digits II–V, whereas metapodiale I resembles the basal phalanges and evidently had a similar function during locomotion. Despite the fact that distale I does not compare morphologically with metapodialia II–V, it might have partially taken over the function of a metapodiale II–V in ray I. In the fossils with a digital arcade in ray I, usually distale I is not only partly in line with distalia II–IV, but also partly aligned with metapodialia II–V. Only in the pes of *Ophiacodon*, and probably also in its manus, does distale I not extend distal to the distalometapodial line.

Therefore, there is a shift in the function of the first ray to one segment more proximal compared to the state in rays II–V: The first phalanx of ray I showed the same function as the penultimate phalanges II–V, metapodiale I was similar to the basal phalanges II–V, and distale I partly overtook the function of metapodialia II–V. This functional shift is often mentioned in the literature; e.g. [1], p. 85: *Titanophoneus*, [8], p. 190: *Tetragonias*, [12], p. 225: Regisauridae indet. CAMZM T 837. Boonstra [40] commented on the manus of Therocephalia: “The metacarpals are well developed – the first is very short and looks very much like the first phalanx…” ([40], p. 130).

In most Synapsida with a digital arcade in ray I, the ratios of the length of the first phalanx I and metapodiale I resemble that of the middle phalanges to the basal phalanx of the lateral digits. Therefore, the proportions of the lever arms in the digital arcade of ray I are similar to those in the lateral digits.

In contrast to non-mammaliamorph Synapsida, the manual and pedal rays I of the analysed Mesozoic Mammaliamorpha do not show a digital arcade, because the first phalanx could be dorsally extended in the metapodiophalangeal joint I. The presence of sesamoids ventral to the head of metapodiale I in the pes of *Zhangheotherium* also suggests the lack of a digital arcade, and indicates instead load transmission through that joint to the substrate [22]. The pes of *?Eucosmodon* and manus or pes of *Eozostrodon* probably possessed sesamoids ventral to the head of metapodiale I as well. In specimens of Mesozoic Mammaliamorpha that are preserved in articulation, the length of the segments of ray I is also a hint for the loss of phalangeal function of the metapodiale: metapodiale I is always shorter than metapodiale II and does not protrude distally to the metapodiophalangeal line, as is the case in non-mammaliamorph Cynodontia (Table 1). Metapodiale I formed a functional unit with the metapodium, which most likely was not the case in non-mammaliamorph Therapsida (*sine* some Anomodontia), where metapodiale I in most taxa is as long as metapodiale II or protrudes distally to the metapodiophalangeal line. Only in some fossil non-mammaliamorph Therapsida is metapodiale I shorter than metapodiale II (Table 1), but in these cases the morphology of the metapodiophalangeal joint clearly shows the presence of a digital arcade.

In the ?manus of *Oligokyphus* (Fig. 16) and in the manus or pes of *Eozostrodon* and *Gobiconodon* (Fig. 17B), metapodiale I shows a morphology intermediate between that of non-mammaliamorph Therapsida and Mesozoic Mammalia. The broad base of metapodiale I prevented an alignment with metapodiale II in the living animal, but was held in an abducted position. In *Oligokyphus*, *Eozostrodon* and *Gobiconodon*, metapodiale I probably was functionally not completely integrated in the metapodium, but separated from it to some degree.

**Figure 23:**
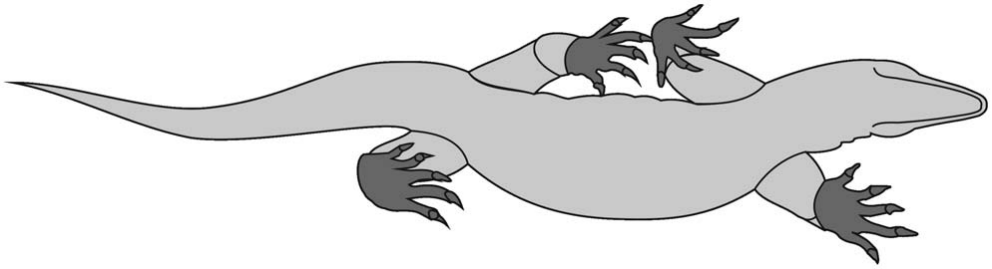
Drawing of a non-therapsid Eupelycosauria with the proportions of *Dimetrodon*. The hypothetical animal was drawn into the tracks of *Dimetropus* from Bromacker, Germany.

The standard position of digit I in Mesozoic Mammaliamorpha equals that of digit I of the manus of *Didelphis* and manus and pes of *Tupaia* ([41], [42], [43] Fig. 6, [23] Fig. 1). In the manus and pes of *Didelphis*, there are also two sesamoids ventral to the head of metapodiale I, which resemble those of the ventral face of metapodialia II–V. A prominent pad is located ventral to the metapodiophalangeal joint I ([38] vol. I, p. 139, [44] Fig. 17, [45] Fig. 16). This pad and the sesamoids show that in *Didelphis* load is transferred through metapodiale I, sesamoids and the pad to the substrate during the propulsion phase. In *Didelphis* and *Tupaia*, metapodiale I is integrated into the metapodium ([38] vol. I, p. 239, vol. II, p. 3). The similarity of the construction of ray I in Mesozoic Mammaliamorpha and the manus of *Didelphis* also is visible in the similar pad distance of ray I in *Ameghinichnus* and the tracks of *Didelphis* in comparison to the pad distance of ray II in the same footprints.

### 2 Rolling Mode in the Propulsion Phase

In mammals with a parasagittal stance, the autopodia roll in the movement plane of the whole extremity. Thus, at the end of the propulsion phase they roll straight in an anterior direction. In the propulsive phase of the sprawling or semi-erect gait, the stylopodium transfers a rotation movement to the zeugopodium, due to its ab- and/or adduction movement (Fig. 23). The zeugopodium then rotates laterally. The zeugopodium itself transfers the rotation to the autopodium. The autopodium either rotates laterally on the substrate, or the rotation must be compensated in the autopodium. Both responses of the autopodium to the rotation of the zeugopodium are called “autopodial rotation”. In the synapsid autopodium, there is hardly any rotation of the autopodium on the substrate. Instead, the rotation of the zeugopodium was compensated by the autopodium during rolling [17]. The compensation of the rotation of the zeugopodium is connected with two different structures: Firstly, rotation could be intercepted at the autopodial joints. This interception takes place through rotation and/or medial abduction in the autopodial joints. Secondly, the rotation of the zeugopodium was compensated by the asymmetric metapodium, which is longer on the lateral than on the medial side (ectaxony). During ventral flexion of the metapodium, this asymmetry leads to rolling of the autopodium in the anteromedial direction, over the proximomedially-distolaterally orientated axis through the basal joints of ray II–IV. At the same time the metapodium (metapodiale II–V) is tilted in the medial direction, and the metapodialia II–V are slightly rotated medially in their basal joints. There is a lateral rotation of the stationary basal phalanges in the basal joints relative to the metapodialia. The medial tilting of the metapodialia II–V during their ventral flexion in the propulsion phase forces the zeugopodium to rotate in lateral direction – provided that the zeugopodium stands more or less perpendicularly to the metapodium and the metapodialia are aligned in the axis of walking at the beginning of the stride [17].

It is thus evident that the mobility of the autopodial joints mirrors the approximate degree of zeugopodial rotation during the propulsion phase if the animal had a mainly terrestrial lifestyle. Therefore, an investigation of the mobility of the autopodial joints allows a reconstruction of the approximate degree of autopodial rotation, which gives a clue about the degree of sprawling possible.

#### Ray I in Propulsion Phase of Non-mammaliamorph Synapsida

In non-mammaliamorph Synapsida, the metapodium is asymmetrical. Therefore, during ventral flexion of the metapodium, the autopodium rolls around the proximomedially- distolaterally orientated, slightly distally convex axis through the basal joints II–IV. In the final stage of ventral flexion of the metapodium, and after its raising, the rolling continues over digit I–II, II–III, II–IV or III–IV, depending on the degree of autopodial rotation in mainly terrestrial animals. If autopodial rotation is high, the rolling ends on digit I. If it is absent, it generally ends on the central digits (III/IV).

As mentioned above, the standard position of the first ray in non-mammaliamorph Synapsida is a digital arcade. In this arcade, force transmission to the substrate took place via the terminal pad and often also over the proximal end of metapodiale I and its underlying pad. In most non-mammaliamorph Synapsida, the proximal end of metapodiale I is situated exactly on the slightly distal convex line through the basal joints of rays II–V (metapodiophalangeal line) in such a way that during ventral flexion of metapodialia II–V the rolling of the autopodium also took place over the proximal part of metapodiale I. Such an arrangement is found in several articulated skeletons; e.g., *Dimetrodon* MNG 10654 (Fig. 5A), *cf. Rubidgea* BP/1/1210 (Fig. 10), *Aelurognathus* SAM-PK-2342, *Glanosuchus* CGS RS424 (Figs. 11A and B), *Microgomphodon* SAM-PK-K10160, and all non-mammaliaform Cynodontia (Figs. 13A, 14 and 15). The proximal part of metapodiale I being in line with the basal joints of ray II–V is also evident in the track record. The proximal imprint of ray I continues proximomedially to the basal pads of rays II–V (e.g. *Dimetropus* and *Gallegosichnus*, Figs. 21A and B). Functionally, the proximal end of metapodiale I also accommodated the load transfer, because the proximoventral convexities likely served as sliding devices to facilitate the mobility of the flexor tendons under the compression load.

The first ray in non-mammaliamorph Synapsida played an important role in the last part of the propulsion phase, because of the rolling of the autopodium in the anteromedial direction. In the evolution toward a parasagittal stance, at least in Cretaceous Mammalia [46], [47], the rolling mode of the autopodium changed from rolling in an anteromedial direction to rolling in the anterior direction. This change is correlated with the mobility of the joints of ray I.

**Figure 24:**
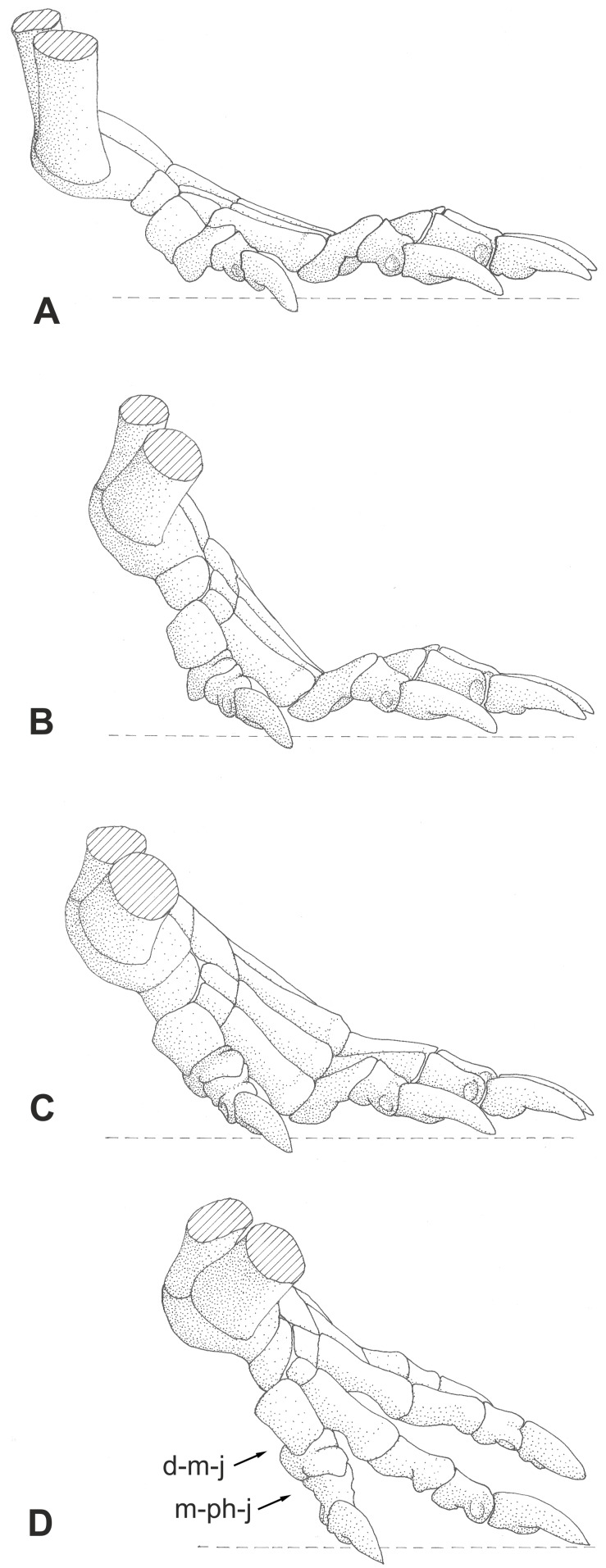
Model of the pedal rolling mode of a *Titanophoneus*-like animal. Approximately middle of the propulsion phase (A), followed by plantar flexion of metatarsalia II–V and distale I (B). C shows the start of the raising phase of the metapodialia and D the start of the raising phase of the digits. Abbreviations: **d-m-j** distalometapodial joint, **m-ph-j** metapodiophalangeal joint.

The reduction of the autopodial rotation in the evolution of Synapsida is seen in the change of the amount of lateral circumduction of metapodiale I in the distalometapodial joint I as well as in the reduced option for a medial abduction and lateral rotation of the first phalanx in the metapodiophalangeal joint I. During ventral flexion of metapodialia II–V and distale I there is a lateral rotation of the basal phalanges with respect to the metapodialia in the basal joints of rays II–V (see above). Accordingly, in the first ray a lateral circumduction of metapodiale I in the distalometapodial joint I must have been possible. This is evidenced by some taxa that were fossilized in articulation (e.g. Figs. 11C and 15). There is no option for a lateral circumduction of metapodiale I in those forms, in which ray I is very short and rolling happened in a straight line with the long axis of distale I and metapodiale I (compare Fig. 24). This means that the medial component in the rolling mode of the autopodium is high. When the rolling occurs in a more anterior direction and ray I is longer than that of *Titanophoneus*, the tilting of the metapodialia and distale I leads to lateral circumduction of metapodiale I relative to distale I. The degree of the transverse arch of the row of distalia also plays a role in the movement of metapodiale I. The higher the curvature of the transverse arch, the more metapodiale I must compensate the curvature by a lateral circumduction movement in order to keep digit I in contact with the substrate during ventral flexion of metapodialia II–V and distale I.

After the elevation of the metapodium off the substrate, and before the elevation of the terminal pads off the substrate, the basipodium and metapodium are rotated laterally along with the zeugopodium. Then the rotation of the zeugopodium was compensated in the digital joints. In this stage of the rolling movement, metapodiophalangeal joint I compensated the rotation of the zeugopodium by medial abduction and lateral rotation of the first phalanx in a way that the terminal pad of digit I could keep contact with the substrate as long as possible (Fig. 24). Therefore, in non-mammaliamorph Synapsida, in which the option of medial abduction and lateral rotation of the first phalanx was high, the autopodial rotation very likely was high, too. In taxa with a more anteriorly directed rolling of the autopodium, these excursion angles were small or even absent.

The rolling mode of ray I will be described for several taxa in more detail.

**Dinocephalian** ***Titanophoneus*** **PIN 157/1, right manus:** In the distalometapodial joint I of *Titanophoneus*, dorsal extension was possible up to 50°, but the joint contact was not as stable as during flexion. Circumduction in the medial direction was possible for about 45°, accompanied by flexion. Circumduction in the lateral direction, as well as lateral abduction, was not possible, and abduction in the medial direction was only slightly permitted.

Phalanx 1 could be flexed for about 70° in the metapodiophalangeal joint I, and an extension up to the zero position was possible. Lateral abduction of about 10° and medial abduction of about 16° could take place, provided the first phalanx was extended. Medial abduction increased during flexion up to 20°, with an inevitable lateral rotation of the first phalanx of about 20°. In the flexed joint, lateral abduction was also feasible, but with a reduced mechanical coherence of the joint.

During ventral flexion of the metapodialia II–V and distale I, metapodiale I was dorsally extended in the distalometapodial joint I up to 50°. In this position, the passive mobility of metapodiale I was high and thus could compensate to some degree for slight abduction or rotation movements of distale I during ventral flexion. The maximum ventral flexion of distale I might have been less than in metapodiale II, because a slight dorsal extension of distale I relative to metapodiale II and distale II in the amphiarthroses of the basipodium may have occurred. Because there is no lateral circumduction possible in the distalometapodial joint I, *Titanophoneus* very likely rolled its autopodium anteromedially, approximately in the direction of the long axis of ray I and II. Consequently, the rolling movement ended over digits I and II (Fig. 24). Thus, during ventral flexion of the metapodialia II–V and distale I, metapodiale I was dorsally extended without much rotation and abduction. After the elevation of the metapodium, when the terminal pads were still in contact with the substrate, the basipodium and metapodium were rotated laterally, together with the zeugopodium. Metapodiale I was then circumducted medially in the distalometapodial joint I. This resulted in phalanx 1 being abducted medially up to an angle of 20°, depending on its degree of flexion in metapodiophalangeal joint I. Phalanx 1 was inevitably also rotated laterally by about 20° in metapodiophalangeal joint I (Fig. 24). In this position, the terminal pad could keep contact with the substrate. The morphological twisting of the distal end in the first phalanx in a lateral direction with respect to its proximal part (Fig. 7), made the lateral rotation of phalanx 1 more effective and thus also supported the terminal pad in keeping contact with the substrate.

The absence of the option for a lateral circumduction of metapodiale I in the distalometapodial joint I and the high degree of medial abduction possible in the metapodiophalangeal joint yield evidence for the high degree of autopodial rotation in ray I in *Titanophoneus*. This is in accordance with the reconstruction of autopodial rotation in the digital joints of ray II–IV [17].

The movement of ray I in ***Dimetrodon*** during the propulsion phase must have been similar to that of *Titanophoneus*, because of the similar joint morphologies seen in the proximal and distal facets of metapodiale I.

**Gorgonopsia:** In the pes of *Gorgonops* BP/1/4089, metapodiale I could be dorsally extended to about 60°, ventrally flexed to about 45°, and medially abducted to approximately 20°. Medial and lateral circumduction was possible to an unknown degree. In the manus of *cf. Rubidgea* BP/1/1210, medial circumduction of more than 35° was possible, and in the manus of gorgonopsian BP/1/600 more than 20°. The metapodiophalangeal joint I is a saddle-shaped joint similar to that of *Titanophoneus*, as was assessed from the slightly damaged metapodiophalangeal joint I of the manus of Gorgonopsia indet. SAM-PK-K4441. The relief of these two joints of ray I is particularly strong in Gorgonopsia, especially in the manus, so that they were very mobile even under high load.

During ventral flexion of metapodialia II–V and distale I, metapodiale I was dorsally extended, probably accompanied by a slight lateral circumduction. During the raising of metapodialia I–IV, flexion and medial circumduction in the distalometapodial joint I took place.

From the option of a slight lateral circumduction of metapodiale I in the distalometapodial joint I, it is concluded that the rolling of the autopodium did not end on rays I+II as in *Titanophoneus*, but probably on rays I+II+III.

**Therocephalia:** In *Theriognathus* NHMUK R 5694, dorsal extension of metapodiale I in the distalometapodial joint I was possible to an unknown degree. Ventral flexion of approximately 10° and medial abduction of about 20° were possible, as well as medial and lateral circumduction of about 20°. The manual distalometapodial joint I of this taxon is morphologically intermediate between the gorgonopsian saddle joint and the pivot joint of Cynodontia.

The metapodiophalangeal joint I of *Theriognathus* NHMUK R 5694 is not typical for Therocephalia, because flexion of the first phalanx shows a very small excursion angle, as far as can be judged from the badly preserved joint. When the joint was extended, medial abduction of phalanx 1 was about 13°, and thus smaller than in *Titanophoneus* in which the angle ranged between 16–20°. During medial abduction the joint contact was mechanically tight and inevitably linked by a lateral circumduction. Abduction in the lateral direction was possible to about 20°, combined with a circumduction of about 10° in a medial direction. Abduction was blocked during maximal flexion.

A high degree of flexion and a medial abduction up to 30° is reconstructed for the metapodiophalangeal joint I of *Ictidosuchoides*, as preserved *in situ* in the articulated skeleton CGS CM 86–655 (Fig. 11C).

The movement of ray I during the propulsion phase was similar to that of Gorgonopsia, except that there was more lateral circumduction of metapodiale I in the distalometapodial joint I during ventral flexion of metapodialia II–V and distale I. In *Theriognathus*, the reduced ability for medial abduction in the metapodiophalangeal joint is a further hint that in comparison to *Titanophoneus*, autopodial rotation was reduced in this taxon.

**Non-mammaliamorph** **Cynodontia:** The distalometapodial joint I in the manus and pes of non-mammaliamorph Cynodontia is most easily described as a pivot joint. Metapodiale I could be laterally and medially circumducted to an unknown degree. The excursion angle was certainly higher in the medial direction than in the lateral, where the latter was constrained by digit II. Lateral circumduction must have been higher than in Gorgonopsia, in which the dorsolateral ridge of distale I is more convex. In contrast, medial abduction of metapodiale I in maximal dorsal extension in the distalometapodial joint I was less possible in Cynodontia than in Gorgonopsia.

The metapodiophalangeal joint I is an ellipsoid joint with three degrees of freedom in the standard and extended position of the first phalanx, but in a highly flexed position it has the character of a hinge joint. Dorsal extension was blocked by a very weak distodorsal protrusion in the earlier Cynodontia or a distally convex lip in the more derived ones.

Rotation of the first phalanx in the metapodiophalangeal joint I is no longer supported by a more coherent match during flexion or extension in this joint. Thus, the mobility of phalanx 1 in the metapodiophalangeal joint I is less constrained than in the other therapsid groups, and it mechanically could react more freely to the movement of the autopodium in the final stage of the propulsion phase.

The increase of lateral circumduction in the distalometapodial joint I and the less constrained mobility in the metapodiophalangeal joint I in comparison with Gorgonopsia indicates a decrease of autopodial rotation in Cynodontia and an increase in the anterior component of the rolling mode. The lateral rotation of the zeugopodium thus must have been reduced in Cynodontia, which is also obvious by studying the digital joints of ray II–V [17].

#### Ray I in Propulsion Phase of Mesozoic Mammaliamorpha

In Mesozoic Mammaliamorpha *(sine Erythrotherium)*, metapodialia II–V are sub-equal in length, which is concluded from articulated autopodial skeletons. In most forms, digits III and IV are nearly equal in length (length difference of metapodiale III and IV is not higher than 4% of the length of metapodiale IV), and in some forms digit III is even longer than digit IV (manus of *Kryptobaatar* and pes of *?Eucosmodon*), or slightly shorter (*Erythrotherium, Megazostrodon* and *Zhangheotherium*). The rolling was brought about approximately in the anterior direction and must have ended over rays III and IV, at least in mainly terrestrial animals. During flexion of the metapodialia, metapodial rotation was completely absent in most forms. It was present to a small degree in the early Mammaliamorpha like *Erythrotherium, Megazostrodon* and *Zhangheotherium*. However, even when metapodial rotation was absent, a slight outward rotation of the zeugopodium still might have been present as far as was compensated in the basal joints, the basipodium and the ankle, wrist and elbow joints.

During the evolution towards a more parasagittal stance and gait, two evolutionary pathways were possible for ray I: 1) the distale and metapodiale of the first ray became longer than in the Therapsida described in this paper, and together act as a support during rolling in an anterior direction. In this case, ray I would have shared a digital arcade with digits II–V. Such a configuration evolved in non-kannemeyeriid Dicynodontia [17]. Alternatively, 2) the distale and metapodiale of the first ray became smaller than in the described non-mammaliamorph Therapsida. Then distale I would have functioned as a part of the row of distalia, and metapodiale I would have been integrated in the metapodium. This is the case in Mesozoic Mammaliamorpha. Thus, there was no digital arcade in ray I in the standard position or during the propulsion phase of Mesozoic Mammaliamorpha (see above).

***Oligokyphus*** **NHMUK R 7515, 7516**: Movement of metapodiale I in the distalometapodial joint I of the early Mammaliamorpha was nearly completely constrained to flexion and extension. If circumduction was possible, it was only to a very small extent. Metapodiale I was always slightly separated from metapodiale II, even in the most adducted position.

In the metapodiophalangeal joint, dorsal extension was possible to an unknown degree, whereas ventral flexion could be brought about to a maximum of 70°. Assuming that the proximal facet of the first phalanx was perpendicular to its shaft, the first phalanx must have been laterally abducted to about 15° in the flexed position. The abduction increased during dorsal extension, inevitably combined with a lateral rotation of the first phalanx. In maximum dorsal extension of the first phalanx, three degrees of freedom very likely were possible.

In the propulsion phase, during ventral flexion of the metapodium (metapodialia I–V), the first phalanx I was dorsally extended, accompanied by an increasing lateral abduction and lateral rotation. Like the lateral rotation of the basal phalanges in the basal joints during ventral flexion of the metapodium, the lateral rotation of the first phalanx in the metapodiophalangeal joint I in *Oligokyphus* was connected to autopodial rotation, which was slightly present in the early forms of Mesozoic Mammaliamorpha (see above). Thus, the terminal pad of ray I could keep contact with the substrate during the entire rolling of the autopodium. In the maximum extended position, the first phalanx very likely had three degrees of freedom and thus could easily passively react to the movement of the basipodium, which might have been of importance after the raising of the metapodium.

In the pedes of *?Eucosmodon* AMNH 16325 and *Kryptobaatar* ZPAL MgM-I/41, the distalometapodial joint I is a saddle joint. The metapodiophalangeal joint in *?Eucosmodon* is similar to that of *Oligokyphus* in having an irregular ball joint dorsally. Its medial face was bigger than the lateral face in dorsal view, so that the distal border of the joint is inclined laterally by about 65° to the shaft of the bone. Therefore, during ventral flexion of the metapodium in the propulsion phase, the first phalanx of digit I might have been laterally abducted and rotated in the metapodiophalangeal joint I as in *Oligokyphus*. Metapodiale I was integrated in the metapodium, in such a way that the saddle shaped distometatarsal joint I in the pedes of *?Eucosmodon* and *Kryptobaatar* did not support the rolling mode on a plane substrate to a high degree, because metapodiale I worked as a unit with the other metapodialia. On an uneven substrate the saddle joint on the basis of metapodiale I might have helped to compensate the irregularities of the surface.

### 3 Opposability of Ray I

Opposition and reposition are combined movements. Rauber and Kopsch [27] describe the opposition movement of the human thumb as follows (translated from German): “Opposition starts by extension and abduction of the thumb; afterwards the thumb makes a combined movement, which is composed of flexion, adduction and inward rotation.” ([27], p. 440).

**Figure 25:**
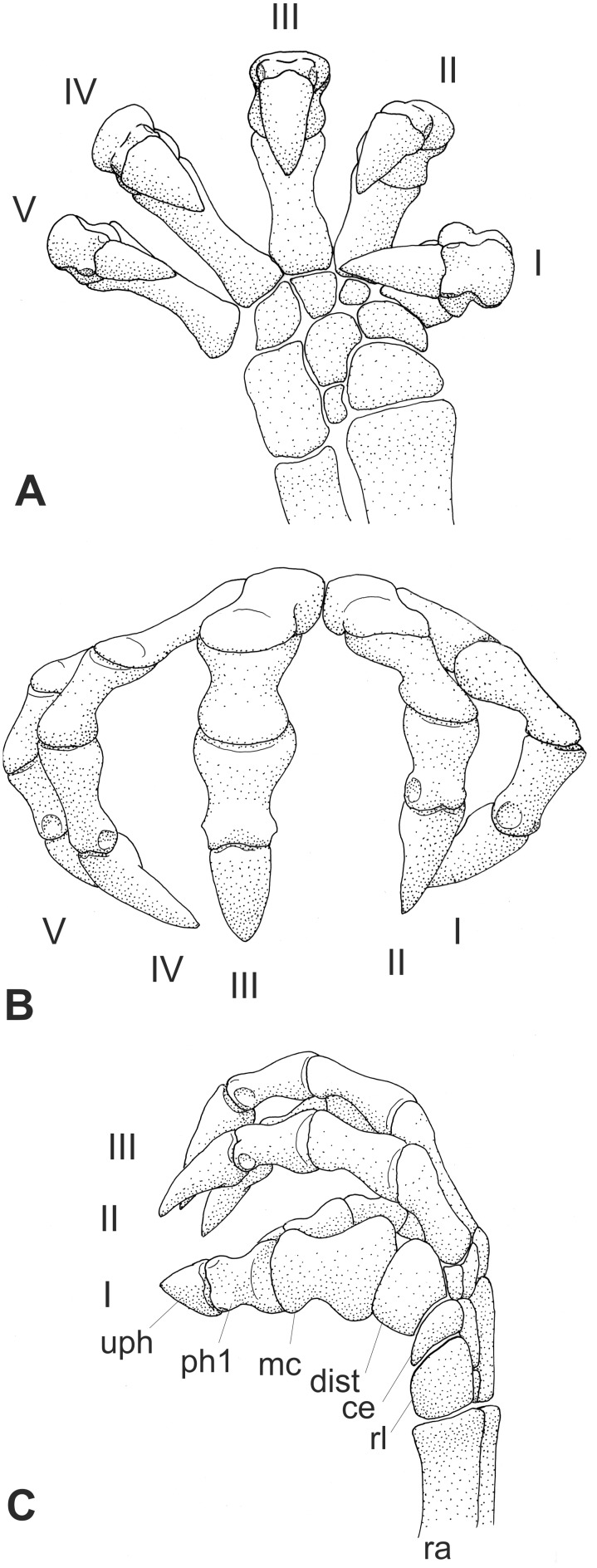
Grasping in a cynodont. The proportions were taken from the manus of *Galesaurus* SAM-PK-K10465. The transverse arch between distale I and distale III was estimated to be 35° and the opposition capacity of ray I against ray III was calculated of about 90°. The gripping manus is seen in palmar (A), anterior (B) and mediodorsal aspect (C). Abbreviations: **ce** centrale, **dist** distale, **mc** metacarpale, **ph** phalanx, **rl** radiale, **ra** radius, **uph** ungual phalanx.

In most Therapsida, metapodiale I showed three degrees of freedom in the distalometapodial joint I: Flexion - extension, ab- and adduction and rotation or circumduction. The opposition movement depends further on the curvature of the transverse arch in the proximal part of the metapodium together with the distal part of the basipodium. When the transverse arch is strongly curved, the tips of the digits can converge distally by flexion of the digits, and less circumduction of ray I is needed for opposition of the first ray. Ray I is tilted against ray III in the transverse arch of the row of distalia and the proximal part of metapodialia in the different therapsid groups; 20° in the phylogenetically early forms *(Biarmosuchus, Titanophoneus* and *Anteosaurus)* and 35–45° in the more morphologically derived forms (gorgonopsian BP/1/4259, Therocephalia and Cynodontia; for more details see Table 2). Therefore, gripping by opposing the first ray and by a high curvature of the transverse arch was possible for most Synapsida (Fig. 25).

**Dinocephalian** ***Titanophoneus*** **PIN 157/1**: *Titanophoneus* had an opposable ray I in manus and pes. All of the excursion angles mentioned in the following text refer to the right manus of the specimen.

In the distalometapodial joint I, medial circumduction of metapodiale I of about 45° was possible and inevitably combined with a slight flexion movement, probably starting in a dorsally extended position. All of this is imperatively aligned with an opposition movement (see above). There was a strong joint contact during flexion and medial circumduction.

The metapodiophalangeal joint I could have taken part in the opposition movement. Flexion of about 70° was possible for the first phalanx I. Lateral abduction and rotation in both directions were possible, but the joint contact during these movements was declined. It appears therefore that the joint could adjust passively to a certain extent to the item, manipulated with the claws.

The mobility of distale I in its amphiarthroses in the basipodium probably further supported the opposition movement of the first ray: “Apparently, digit I can be moved in medial direction and, to a certain degree, opposed against the others, [a movement] resulting from the mobility of ray I in the amphiarthroses of centrale I and carpale distale I” (translated from Russian, [1], p. 86).

Thus, the excursion path during the opposition of ray I is composed of the excursion of several segments in their respective joints. The transverse arch formed by the row of distalia, and the proximal portion of metapodialia participated roughly with 20°. This 20° together with the opposition movement in the distalometapodial joint I (45°) comprised 65°. The other joints and amphiarthroses probably added 10° to the degree of the opposition movement. Adding all anatomical facts together an opposition of ray I in the manus of *Titanophoneus* was possible, during which a circumduction of about 75° of the terminus of ray I in relation to ray III occurred.

Only a small amount of abduction was possible in the distalometapodial joint I, so the abduction needed for the beginning of the opposition movement must have taken place in the centralodistal joint I. The medial part of the articular face of the medial centrale with its ellipsoidal joint surface shows that there was the option for abduction of distale I in the centralodistal joint I.

Compared with most other Therapsida, the rugose process on the proximomedial side of metapodiale I is very prominent in *Titanophoneus.* This suggests that the abductor, flexor and extensor tendons inserting there were strong, another indicator of free mobility of ray I in *Titanophoneus*.

Ray I in the pes had nearly the same opposition capacity as that of the manus. Only the excursion angles were a little smaller, due to the lower relief of the joints.

**Eupelycosaurian** ***Dimetrodon***: As far as can be judged from the investigated material, there was at least the option for an opposition movement of ray I in *Dimetrodon*, which was very similar to *Titanophoneus*. Only the excursion angles might have been a little smaller given the lower relief as compared to *Titanophoneus*.

**Gorgonopsia:** Circumduction of metapodiale I in a medial direction in the distalometapodial joint I could have taken place. In the manual distalometapodial joint I of gorgonopsians BP/1/4259, BP/1/600, CGS CM 86–471 and *cf. Rubidgea* BP/1/1210, the capacity of circumduction of metapodiale I must have been greater than in the pes of *Gorgonops* BP/1/4089 and gorgonopsian SAM-PK-K4441, because of the higher relief of their joints. In *cf. Rubidgea* BP/1/1210, medial rotation of metapodiale I of about 35° is preserved *in situ*. The maximum circumduction probably reached an angle of 45°, like in *Titanophoneus*. In addition to medial circumduction, medial abduction was an option for metapodiale I in the distalometapodial joint I in Gorgonopsia. In the pes of *Gorgonops* BP/1/4089, the maximum medial abduction angle was about 20°. The main abduction, which was possible in ray I, might have taken place in the centralodistal joint I, as is the case in *Titanophoneus*.

The metapodiophalangeal joint is a saddle joint similar to that of *Titanophoneus*. Consequently, the mobility of the first phalanx in that joint was also similar.

In the manus of *cf. Rubidgea* BP/1/1210, and the gorgonopsians BP/1/4259 and BP/1/600, a strong relief characterizes the surfaces of all joints in the first ray. The entire opposition movement of digit I is reconstructed as follows: The transverse arch added around 35° to the entire excursion angle, the amphiarthroses of the carpalia about 5°, the distalometapodial joint I about 45° and the metapodiophalangeal joint I 5° or more. In total, ray I could be opposed with a circumduction angle of about 90° in relation to ray III. In the pes examined, it must have been less than 90°, because of the lower relief than that of the manus.

During opposition, medial abduction took place in the centralodistal joint and in the distalometapodial joint I and probably some lateral abduction occurred in the metapodiophalangeal joint I as well. Flexion of metapodiale I took place in the distalometapodial joint I, starting from a dorsally extended position of metapodiale I. The main flexion movement was added in the metapodiophalangeal and the terminal joint.

As in *Titanophoneus*, the rugose process at the proximomedial part of metapodiale I is very prominent in the manus or pes of *?Gorgonops* SAM-PK-K7580 and in the manus of gorgonopsians BP/1/4259 and BP/1/600. This process suggests the presence of strong abductor, flexor and extensor tendons.

**Therocephalian** ***Theriognathus*** **NHMUK R 5694 (manus)**: Medial circumduction of metapodiale I resulted in an abduction of approximately 20°. Simultaneously, a medial circumduction of approximately 20° could be brought about and a flexion of metapodiale I up to zero position was possible. The metapodiophalangeal joint I supported the opposition movement by flexion, probably combined with some lateral abduction and medial rotation. However, rotation and abduction was blocked during maximal flexion.

In the amphiarthroses and joints connecting distale I with the other carpalia, an unknown amount of abduction was possible.

The transverse arch added about 30° to the opposition movement of digit I against digit III, the distalometapodial joint 20° and the amphiarthroses probably 5° or more. So an opposition of about 60° against ray III could have occurred.

**Other Therocephalia**: The distalometapodial joint I of *Glanosuchus* is similar to that of Gorgonopsia. Therefore, the opposition movement likely resembled that of Gorgonopsia. In *Glanosuchus*, there seems to be no substantial difference between manus and pes.

An at least partly opposable ray I in manus and pes of *Ericiolacerta parva* CAMZM T 369 is reported by Watson [39]: “…. the thumb and big toe possessed some independence and were movable as units; indeed, it is not inconceivable that they may have been in some degree ‘opposable’.” ([39], p. 1178).

**Cynodontian** ***Galesaurus*** **SAM-PK-K10468 (manus),** ***Thrinaxodon*** BP/1/**2776 (manus)** **and** ***?Scalenodon*** **NHMUK R 9391 (pes)**: In the distalometapodial joint I, a pivot joint, the ability of medial abduction was probably less expressed than in Gorgonopsia. But because the joint contact was feeble, a small amount of medial abduction might have been possible in the standard position. In a joint with a high relief like that of *?Scalenodon*, a medial circumduction of about 45° is tentatively estimated.

In *?Scalenodon*, the capacity of ventral flexion of phalanx 1 in the metapodiophalangeal joint I is estimated at around 90°. Due to the open articular surface of the head of metapodiale I, there was an option for a slight rotation of phalanx 1 in the metapodiophalangeal joint I. In full flexion, however, both abduction and rotation were blocked. Therefore, during the opposition movement the first phalanx was flexed, but the amount of rotation and abduction possible in that joint depended on the degree of flexion.

The transverse arch in *?Scalenodon* contributed with about 35° to the opposition movement of ray I. About 45° was added by circumduction possible in the distalometapodial joint I. All other joints of ray I added probably about 10°, so that the opposition capacity of ray I in the pes of *?Scalenodon* was about 90° against ray III.

**Mesozoic Mammaliamorpha**: After the shift of metapodiale I from a functionally phalangeal to a functionally metapodial element at the transition from non-mammaliamorph Therapsida to Mammalia, the freedom of abduction of metapodiale I and its opposition capacity was not necessarily lost. Medial circumduction in the distalometapodial joint was no longer an obligatory component of autopodial rotation, but this movement could persist or re-evolve in some animals with grasping abilities. In *Homo sapiens*, for instance, ray I in the manus is opposable, though metacarpale I is fully integrated in the metacarpus.

According to the roller shaped proximal facet of metapodiale I of the ?manus of *Oligokyphus* and manus or pes of *Eozostrodon*, an opposition movement of metapodiale I in the distalometapodial joint I was not likely, and if there was any option for that, the degree must have been extremely small. The first phalanx was slightly laterally abducted in the metapodiophalangeal joint I of 15° in the maximal flexed position. During ventral flexion of the first phalanx, inevitably a slight medial rotation took place. Both lateral abduction and medial rotation of the first phalanx facilitated opposition of the first ray, but the excursion angle is not big enough to suggest an opposable ray I.

For *Zhangheotherium* IVPP V7466, Hu et al. [16] state the following: “The arrangement of the carpal elements in *Zhangheotherium* is similar to that in *Asioryctes* (…). Like *Asioryctes*, *Zhangheotherium* has a ‘spreizhand’ typical for living generalized insectivores and rodents. (…) The pollex and the digit V are somewhat separate from other digits, but there is no evidence supporting opposable pollex and grasping ability” ([16], p. 121). The degree of the possible spread of the digits must be reconstructed in order to know the degree of prehensility, which would be possible just by flexing the digits. Such a form of prehensility was probably very high in some Mesozoic Mammaliamorpha, as is suggested by the Jurassic ichnotaxon *Ameghinichnus*. Manual rays I and V diverge by about 115° and pedal rays I and V by about 150° (Fig. 22A).

In the manus or pes of *Gobiconodon* MCZ 19860 and the pedes of *?Eucosmodon* AMNH 16325 and *Kryptobaatar* ZPAL MgM-I/41 the distalometapodial joint I is a saddle joint. According to the nomenclature of Kielan-Jaworowska and Gambaryan [13], however, this joint in the pes of *Kryptobaatar* is a hinge joint. Granger and Simpson [14] suggest that a small amount of opposability of metapodiale I was possible in the pes of *?Eucosmodon*. Jenkins and Schaff [48] depict metapodiale I in the following way: “In two metapodials (?pollical, ?hallucal) the proximal articular surfaces are saddle-shaped and thus raise the possibility of some independent movement of the pollex or hallux.” ([48], p. 20). Kielan-Jaworowska and Gambaryan [13] describe the movement in the distalometapodial joint I of the pes of *Kryptobaatar* as follows: “The joint between the metatarsale I and entocuneiform is of a hinge type and permits movements of Mt I in a dorsoplantar direction; it seems, however, that a very small abduction was also possible.” ([13], p. 80).

In order to understand the motion options of the first ray in climbers like *Sinodelphys* and *Eomaia*, where a higher mobility of ray I than in terrestrial Mesozoic Mammaliamorpha is likely, the joints must be micro-CT scanned for a detailed analysis.

### 4 Grasp Capacity of Manus and Pes

An opposable first ray is a prerequisite for a grasping movement amongst Synapsida. There are further structures of the autopodium that support the assumption of prehensility. Saddle-shaped structures in the distalometapodial joints of ray II and V could presumably have added to the prehensility. Gorgonopsia in particular show a pronounced relief in the distalometapodial joint of ray II. The transverse arch of the distal part of the basipodium together with the proximal part of the metapodium and its option for prehensility was already mentioned in the last section.

The opposition of ray I, the transverse arch and probable saddle-shaped structures in the distalometapodial joints of ray II and V resulted in the convergence of the digital tips during grasping (Fig. 25). Because of this there is no necessity for the digital joints of ray II–V to contribute to rotation and abduction in prehensility. The capacity of abduction and rotation of the digital joints of digits II–V, by contrast, is reduced during flexion. In the fully flexed position, abduction was blocked in such a way that only a minute amount of rotation was possible. This resulted in a mechanically coherent match of the joint surfaces during flexion. The digital joints in rays II–V were very stable against torsion and shear in the flexed position resulting in a stable and powerful grip [17].

Only a few specimens of fossil Synapsida preserve both manus and pes on the same animal. In those cases, manus and pes show about the same capacity of grasping as described above. In *Titanophoneus* the distalometapodial, as well as the metapodiophalangeal joint of ray I shows stronger relief in the manus than in the pes. Because the naviculare is not preserved, the mobility of distale I in manus and pes cannot be compared. In the gorgonopsian SAM-PK-K4441, the excursion angles of ray I were higher in the manus than in the pes, as far as can be judged from dorsal aspect of the articular gap. In the other Gorgonopsia, pedes and manus are not preserved in the same specimen, but judging from the single manus and pedes of gorgonopsian specimens, the manus usually shows a stronger articular relief than the pes. In *Glanosuchus* the distalometapodial joint of manus and pes are coincident in dorsal view. The pronounced relief of the pivot-shaped distalometapodial joint I in *?Scalenodon* shows that also in the pedes of Cynodontia, a grasp capacity with an opposable ray I has been present. Apparently, there was a general grasp capacity in the manus and pes of non-mammaliamorph Therapsida, with some differences in the degree of prehensility, opposability and the degree of curvature of the transverse arch. However, this does not imply that all of them used their autopodia for manipulation, because medial circumduction of ray I also helped in the rolling of the autopodia. In conclusion, some animals may have used medial circumduction of metapodiale I in the distalometapodial joint I just for rolling at the end of the propulsion phase after the raising of the metapodium and not for grasping movements. In Gorgonopsia (*sine* gorgonopsian SAM-PK-K4441) and non-mammaliamorph Cynodontia, however, the capacity of opposability of metapodiale I in the distalometapodial joint I is as high as in *Titanophoneus*, despite the fact that autopodial rotation is smaller. This would mean that grasping and manipulating was so commonly used in Gorgonopsia and non-mammaliamorph Cynodontia that these structures persisted within these groups. In Gorgonopsia the highly mobile distalometapodial joint of ray II is also a hint that grasping belonged to the operational repertoire of manus and pes.

In the manus and pes of *Titanophoneus*, and in the manus of *Theriognathus* and the gorgonopsian SAM-PK-K4441, the grasping ability was less developed than in the manus of most Gorgonopsia and non-mammaliamorph Cynodontia investigated in this study. In the former taxa it appears likely that the construction mainly supported the rolling mode of ray I without contradicting a grasping option, e.g., for handling prey or digging.

Because of their grasp capacity carnivorous non-mammaliamorph Therapsida, e.g., Gorgonopsia, could hold their prey with the manus and pedes. This holding of the prey was a prerequisite for the development of large, laterally compressed fangs (saber teeth) that mainly take loads in the longitudinal direction. Lateral forces, e.g., those applied by struggling prey, could have resulted in failure of the tooth [49].

## Conclusions

In seventy-eight Early Permian to Late Cretaceous non-anomodont Synapsida, the range of motion of the manual and pedal ray I is reconstructed, based mainly on the analysis of the joints of ray I.

The standard position of ray I in non-mammaliamorph Therapsida and *Dimetrodon* is a digital arcade in the metapodiophalangeal joint I. Amongst Anomodontia, some forms secondarily lost the digital arcade in the first ray, but these are not within the frame of this paper. In all other non-mammaliamorph Therapsida the distodorsal part of the distal facet on metapodiale I bears a distally pointing protrusion or a distally curved lip, which prevented phalanx 1 from dorsal extension. In an intermediate position of phalanx 1 in the metapodiophalangeal joint I, the respective joint facets show an area of reduced curvature and a good fit. Therefore, it appears very likely that this position refers to the preferred configuration of the bones in the living animal: the standard position. In this position the flexion of phalanx 1 varies between 10° and 45° (manus of *Titanophoneus* PIN 157/1: ∼25°, gorgonopsian SAM-PK-K4441: ∼20°, *Glanosuchus* CGS RS424: ∼35°, *Theriognathus* NHMUK R 5694: ∼10° (?), *Galesaurus* SAM-PK-K10468: ∼45°, *Thrinaxodon* BP/1/2776 and BP/1/1737: ∼40° and pes of *Dimetrodon* MNG 10654: ∼40° and *Gorgonops* BP/1/4089: ∼30°).

In contrast to the first ray of non-mammaliamorph Therapsida and *Dimetrodon*, the digital arcade of digits II–V was not formed by the metapodiophalangeal joints, but by the middle joints. This means that the digital arcade in ray I is shifted to one segment more proximal than in the rays II to V: Form and function of phalanx 1 of ray I is very similar to that of the middle phalanges of the digits II–V and metapodiale I resembles the basal phalanges of digits II–V in form and function. Distale I is functionally similar to the metapodialia II–V, but not referring to the morphology of the bone, which is rectangular and not hourglass-shaped as in metapodialia II–V.

In Mesozoic Mammaliamorpha, however, a digital arcade in the metapodiophalangeal joint I was absent. Phalanx 1 was dorsally extendable in the metapodiophalangeal joint I and metapodiale I functionally formed part of the metapodium. In *Oligokyphus* and *Eozostrodon* metapodiale I probably was not completely integrated in the metapodium, which is concluded from the very wide proximal end of the bone. In Mesozoic Mammalia, however, metapodiale I was aligned to metapodiale II and forms a functional unit with metapodialia II–V at least during terrestrial locomotion.

Ichnology also yields an indication of the orientation of the bones of ray I in the living animal. In tracks referred to non-mammaliamorph synapsid trackmakers, the imprint of the terminal pad and the proximal pad or sole cushion has the same distance in both ray I and ray II. This strongly suggests a similar morphology and function of ray I (metapodiale plus phalanges 1 and 2) and digit II (phalanges 1–3). In the mammal track *Ameghinichnus*, ray I is shorter and shows a length of ∼67% and ∼77% of digit II in two manual and ∼71% and ∼90% in two pedal imprints. This yields evidence for the shift of the phalangeal to the metapodial function of metapodiale I in Mesozoic Mammaliamorpha.

Animals with an abducted limb posture usually show autopodial rotation during the propulsion phase. During autopodial rotation in Synapsida, the rotation transferred from the zeugopodium into the autopodium during rolling is compensated in the autopodial joints and by metapodial rotation, and thus allows a stable contact of the digital pads with the substrate and avoids rotational friction on the ground. As is judged from the mobility in the joints of ray I, autopodial rotation could have been high, when both the angle of lateral rotation of the first phalanx and that of its medial abduction in the metapodiophalangeal joint I was large. The reduction of these angles is congruent with the decrease of autopodial rotation. In non-mammaliamorph Synapsida, with their highly mobile metapodiale I, evidence for the reduction of autopodial rotation is also provided by the increase of lateral circumduction that was possible in the distalometapodial joint I. Therefore, based on ray I, a gradual decrease of autopodial rotation I was reconstructed for Synapsida in the following order: *Titanophoneus* – Gorgonopsia – Therocephalia – basal Cynodontia *(Procynosuchus, Galesaurus)* – derived Cynodontia *(Thrinaxodon, Diademodon, ?Scalenodon)* – basal Mammaliamorpha (*Oligokyphus*, Morganucodontidae) – Mammalia.

Many Synapsida showed grasping capacities, which are mainly based on an opposable ray I and the curvature of the transversal arch of the row of distalia and the proximal part of metapodialia. The option for a medial circumduction of metapodiale I in the distalometapodial joint I is an essential prerequisite for an opposable ray I. The metapodiophalangeal joint I could have contributed slightly to the opposition movement of ray I by a medial circumduction of phalanx 1, as well as the amphiarthroses, which connect distale I with the bones of the carpus or tarsus, respectively. Due to the curvature of the transverse arch, the digits converge with each other just by flexing the digital joints. Like this the curvature of the transverse arch supports the opposition movement of ray I.

Bringing all anatomical facts together, the possible opposition of the terminus of ray I in relation to ray III was reconstructed in Synapsida to be ∼75° in the manus of *Titanophoneus*, ∼90° in the manus of *cf. Rubidgea* BP/1/1210 and the gorgonopsians BP/1/4259 and BP/1/600, ∼60° in the manus *Theriognathus* NHMUK R 5694 and ∼90° in the pes of *?Scalenodon* NHMUK R 9391. Within Mesozoic Mammaliamorpha, a relatively high mobility of metapodiale I was present in the manus or pes of *Gobiconodon* and in the pes of *?Eucosmodon* and *Kryptobaatar* [48], [14], [13]. The amount of opposability of ray I, if any, remains unclear.

Besides an opposable ray I and a strongly curved transverse arch, saddle-shaped structures in the distalometapodial joint II and V improved the grip capacity in Synapsida. In Gorgonopsia, these structures are most evolved among the Synapsida analysed in this study. During flexion the digital joints of digit II–V in all Synapsida show stable joint contacts with no freedom of abduction and, if at all, little freedom of rotation. Therefore, the digital joints in rays II–V of Synapsida were very stable against torsion and shear during gripping. Usually the grip capacity was more pronounced in the manus than in the pes, but fossils like *?Scalenodon* show that a pronounced grasping capacity could have been present in the pes as well.

The morphology of the joints of ray I shows a general grasping capacity in the manus and pes of non-mammaliamorph Therapsida, with some differences in the effectiveness of gripping. However, medial circumduction of metapodiale I in the distalometapodial joint I was obligatory in the rolling mode of non-mammaliamorph Therapsida, and therefore is no unequivocal indication of the presence of a grasping option. However, in the carnivorous group Gorgonopsia (*sine* gorgonopsian SAM-PK-K4441) and in non-mammaliamorph Cynodontia, which were partly carnivorous and partly omnivorous, medial circumduction of metapodiale I is as high as in *Titanophoneus*, although the autopodial rotation is smaller. Because of this, grasping and manipulating items must have been very common within these two groups, so that the relevant structures persisted within them.

The grasping ability of carnivorous Therapsida may have played a role in fixing the prey during the biting and tearing and might also have helped to prevent breakage of the laterally compressed fangs.

## Supporting Information

S1 Table **Measurements on metapodiale I and phalanges of digit I and II in manus and pes of Early Permian to Late Cretaceous non-anomodont Synapsida.**
(XLSX)
